# Immune defects associated with lower SARS-CoV-2 BNT162b2 mRNA vaccine response in aged people

**DOI:** 10.1172/jci.insight.161045

**Published:** 2022-09-08

**Authors:** Joana Vitallé, Alberto Pérez-Gómez, Francisco José Ostos, Carmen Gasca-Capote, María Reyes Jiménez-León, Sara Bachiller, Inmaculada Rivas-Jeremías, Maria del Mar Silva-Sánchez, Anabel M. Ruiz-Mateos, María Ángeles Martín-Sánchez, Luis Fernando López-Cortes, Mohammed Rafii-El-Idrissi Benhnia, Ezequiel Ruiz-Mateos

**Affiliations:** 1 Clinical Unit of Infectious Diseases, Microbiology, and Preventive Medicine, Institute of Biomedicine of Seville (IBiS), Virgen del Rocío University Hospital, Spanish Research Council (CSIC), and; 2 Department of Medical Biochemistry, Molecular Biology, and Immunology, School of Medicine, University of Seville, Seville, Spain.; 3 Centro de Salud Pinillo Chico, El Puerto de Santa María, Spain.

**Keywords:** Immunology, Vaccines, Adaptive immunity, Cellular senescence, Innate immunity

## Abstract

The immune factors associated with impaired SARS-CoV-2 vaccine response in elderly people are mostly unknown. We studied individuals older than 60 and younger than 60 years, who had been vaccinated with SARS-CoV-2 BNT162b2 mRNA, before and after the first and second dose. Aging was associated with a lower anti–RBD IgG levels and a decreased magnitude and polyfunctionality of SARS-CoV-2–specific T cell response. The dramatic decrease in thymic function in people > 60 years, which fueled alteration in T cell homeostasis, and their lower CD161^+^ T cell levels were associated with decreased T cell response 2 months after vaccination. Additionally, deficient DC homing, activation, and TLR-mediated function, along with a proinflammatory functional profile in monocytes, were observed in the > 60-year-old group, which was also related to lower specific T cell response after vaccination. These findings might be relevant for the improvement of the current vaccination strategies and for the development of new vaccine prototypes.

## Graphical abstract

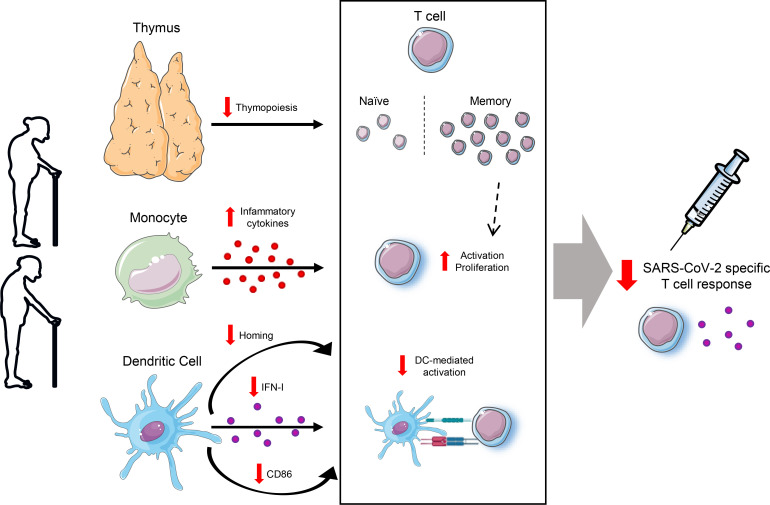

## Introduction

Immune aging is sustained by multifaceted remodeling of innate and adaptive immunity, and it includes a diminished response to new antigens, a decreased memory T cell response, and a persistent chronic inflammation (1–4). Immune aging leads to more severe consequences of viral infections, as well as lower protection following vaccination (5). SARS-CoV-2 infection and its associated disease, COVID-19, are known to have a higher impact in aged people. In fact, delayed viral clearance, prolonged disease, and higher COVID-19 fatality rate have been related to age (6), and approximately 80% of hospitalizations involved people older than 65 years (7, 8).

Vaccination is the most effective tool for the prevention of the serious symptomatology caused by SARS-CoV-2 and other viral infections, especially for vulnerable populations such as elderly people (9, 10). The BNT162b2 mRNA vaccine, commonly known as the Biontech/Pfizer vaccine, has shown high safety and efficacy against severe outcomes of COVID-19 (11). The 2-dose vaccination of this SARS-CoV-2 vaccine induces a strong humoral response measured by the magnitude of binding antibodies to coronavirus Spike (S) protein and the neutralization capacity of the antibodies (11, 12). In addition, notable SARS-CoV-2 S–specific CD4^+^ and CD8^+^ T cell responses have been observed after BNT162b2 vaccination (13–15).

In spite of the promising results of the BNT162b2 vaccination, a lower effectiveness — in terms of COVID-19 symptoms, admissions to hospital, and deaths after the vaccination — has been reported in elderly people (16–18). Moreover, other studies have described lower levels of neutralizing antibodies in vaccinated elderly people compared with younger participants (19–21), especially 6 months after the second dose (22, 23). In addition, low levels of S-specific T cell response after vaccination have been shown in the elderly (20). However, the adaptive and innate immune factors associated with the lower vaccine response in elderly people are not yet characterized.

A better understanding of the age-related immune dysfunction to the SARS-CoV-2 vaccine is crucial for future vaccination strategies to improve older adults’ protection against this virus. Thus, the aim of this study was to investigate the major immune alterations in aged people, in terms of both SARS-CoV-2–specific adaptive and innate immunity, associated with a lower response to the SARS-CoV-2 BNT162b2 mRNA vaccine.

## Results

### Association of SARS-CoV-2–specific IgG levels with age.

In this study, we included 54 healthy adults vaccinated with the BNT162b2 mRNA vaccine against SARS-CoV-2, classified according to their age: 33 young people < 60 years of age (median, 29 years [interquartile range (IQR) 26–49 years]) and 21 aged people > 60 years of age (median 73 years [IQR 72–74 years]). Comorbidities of all donors are presented in Supplemental Figure 1 (supplemental material available online with this article; https://doi.org/10.1172/jci.insight.161045DS1). As it was expected, the aged group showed a higher percentage of donors with comorbidities, with cardiovascular diseases and arthrosis appearing most prominently (Supplemental Figure 1). Three participants were excluded from the study due to a positive result for SARS-CoV-2 RNA PCR or antibodies against receptor-binding domain (RBD) of the S protein of SARS-CoV-2 prior to vaccination. The innate and adaptive immunity parameters were analyzed before vaccination (PRE); 3 weeks after the first dose, just before the administration of the second dose (1D); and 2 months after the second dose (2D) (Supplemental Figure 1).

**Figure 1:**
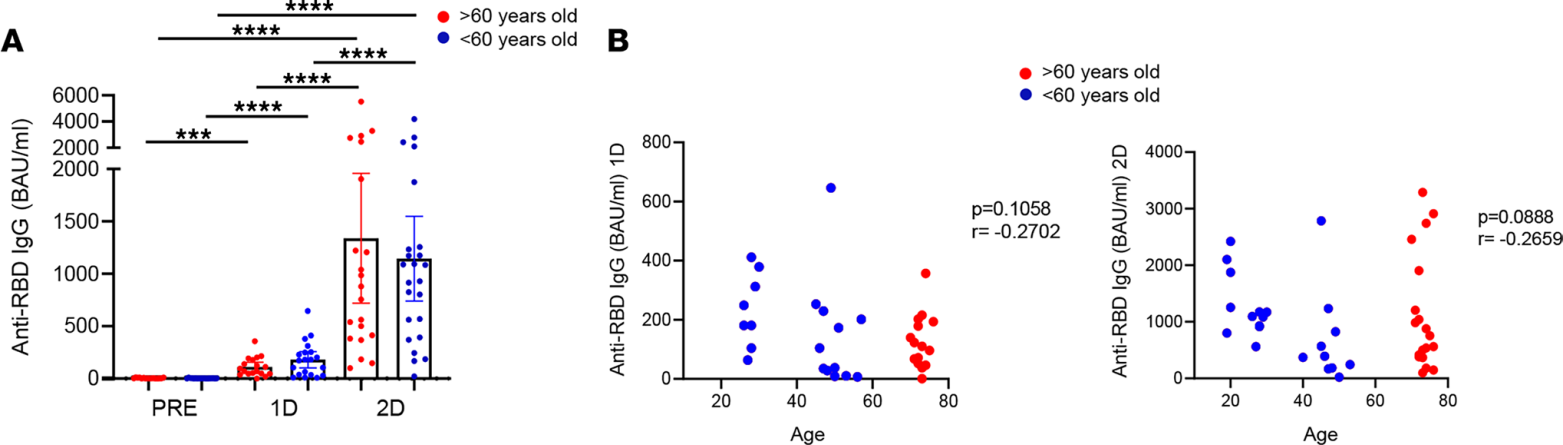
Association of SARS-CoV-2–specific IgG levels with age. (**A**) Anti–RBD IgG levels (binding antibody units [BAU]/mL) in participants > 60 years old (red) and those < 60 years old (blue) before SARS-CoV-2 vaccination (PRE), 3 weeks after the first dose (1D), and 2 months after the second dose (2D). (**B**) Correlation of anti–RBD IgG levels with age in all the study participants after the first dose (left) and after the second dose (right). Mann-Whitney *U*, Wilcoxon, and Spearman tests were used (*n =* 54). Friedman test was applied in **A** (>60-year-old, ****P ≤ 0.0001; <60-year-old, ****P ≤ 0.0001).

Firstly, anti–RBD SARS-CoV-2 IgG levels were quantified by RBD-specific ELISA in > 60-year-old and < 60-year-old people at the 3 time points described above. In accordance to previous studies (11, 20), the BNT162b2 mRNA vaccine induced the production of SARS-CoV-2–specific IgG levels, and these levels were much higher after the second dose compared with the first dose (Figure 1A). Although no significant differences were observed between aged and young participants, young people tended to produce higher levels of specific antibodies after the first dose (Figure 1A). This tendency was also observed when we correlated anti–RBD IgG levels and age after the administration of the first dose (Figure 1B, left) and after the second dose (Figure 1B, right). In addition to SARS-CoV-2–specific antibodies, we also determined IgG autoantibodies against IFN-α in plasma of the studied donors. In fact, anti–IFN-Is have been previously observed in severe COVID-19 patients (24), but the role of these antibodies in the context of SARS-CoV-2 vaccination remains unknown. anti–IFN-α IgGs were observed only in 2 donors; these autoantibodies were found before and after SARS-CoV-2 vaccination (Supplemental Figure 2). Therefore, only 2 donors were positive for anti–IFN-α autoantibodies, and this was not related to the response to SARS-CoV-2 vaccination.

### Aged people show a lower and less polyfunctional SARS-CoV-2 S–specific CD4^+^ and CD8^+^ T cell response after vaccination.

**Figure 2:**
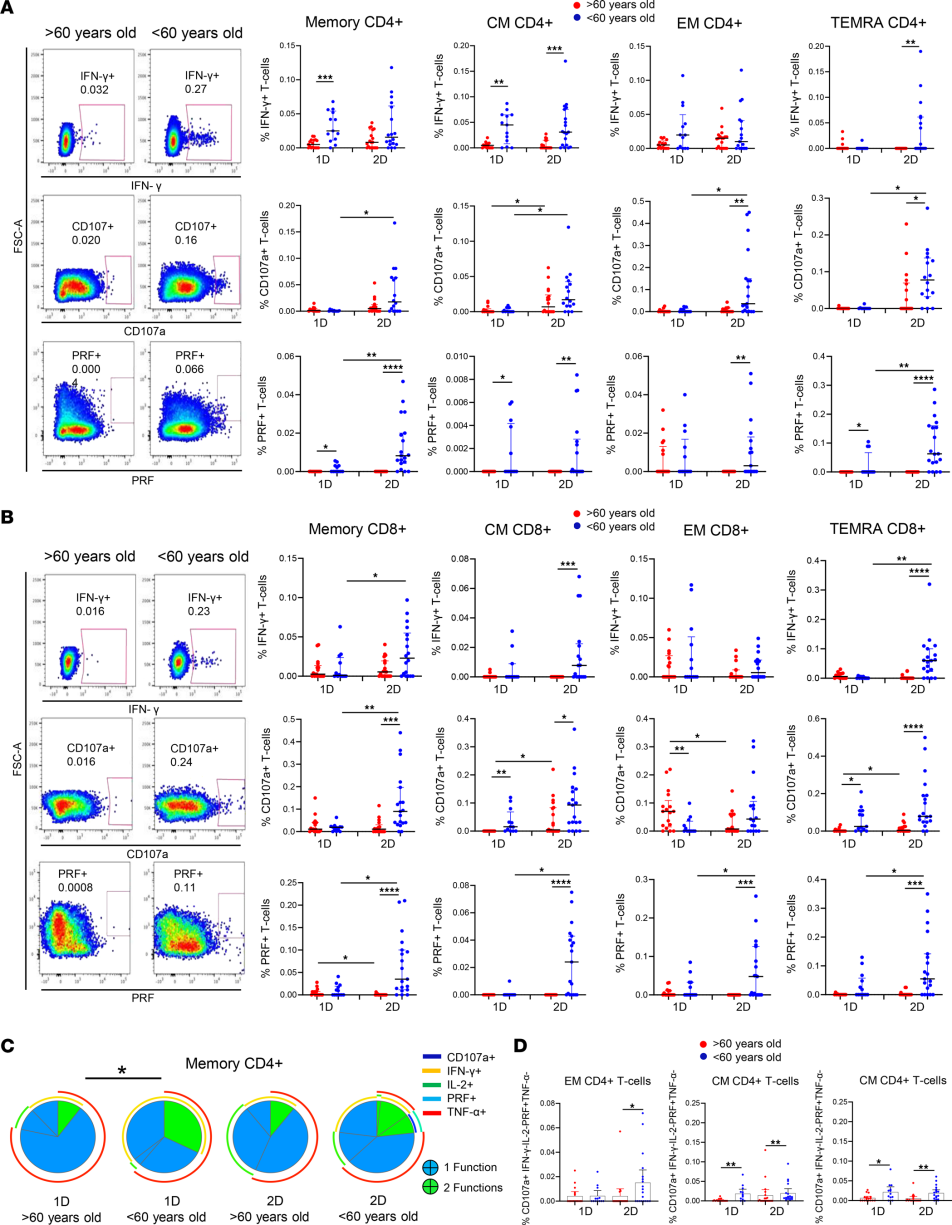
Aged people show a lower and less polyfunctional SARS-CoV-2 S–specific CD4^+^ and CD8^+^ T cell response after the vaccination. (**A** and **B**) Dot plots presenting the percentage of IFN-γ^+^, CD107a^+^, and PRF^+^ cells within memory, CM, EM, and TEMRA CD4^+^ (**A**) and CD8^+^ (**B**) T cells after S-specific SARS-CoV-2 stimulation, comparing participants > 60 years old (red) and < 60 years old (blue) 3 weeks after the first dose (1D) and 2 months after the second dose (2D) of SARS-CoV-2 vaccine (right). Pseudocolor dot plot graphs show a representative data of memory CD4^+^ T cells from a > 60-year-old and a < 60-year-old donor 2 months after vaccination (left). (**C**) Pie charts representing SARS-CoV-2 S–specific memory CD4^+^ T cell polyfunctionality. Each sector represents the proportion of S-specific CD4^+^ T cells producing 2 (green) or 1 (blue) functions. Arcs represent the type of function (CD107a, IFN-γ, IL-2, PRF, and TNF-α) expressed in each sector. (**D**) Bar graphs showing the percentage of EM and CM CD4^+^ T cells expressing different combinations of studied functions (CD107a, IFN-γ, IL-2, PRF, and TNF-α) comparing > 60-year-old (red) and < 60-year-old (blue) participants after the first (1D) and the second (2D) dose. Mann-Whitney *U* (**A**, **B**, and **D**), Wilcoxon (**A**, **B**, and **D**), and Permutation (**C**) tests were used (*n =* 41). *P < 0.05, **P < 0.01, ***P < 0.001, ****P < 0.0001.

We investigated the magnitude and polyfunctionality of SARS-CoV-2 S–specific CD4^+^ and CD8^+^ T cell response through intracellular cytokine staining. We analyzed these parameters in total memory (Memory), central memory (CM), effector memory (EM), and terminal differentiated effector memory (TEMRA) CD4^+^ and CD8^+^ T cells (Supplemental Figure 2A). Three weeks after the administration of the first dose, CD4^+^ T cells principally produced IFN-γ and TNF-α as acute responses to SARS-CoV-2 S protein, but they also expressed low levels of the degranulation marker CD107a, perforin (PRF), and IL-2 (Supplemental Figure 2B). Importantly, CD4^+^ T cells from people > 60 years old showed a lower SARS-CoV-2 S–specific IFN-γ production and cytotoxic response, reflected in the percentage of CD107a^+^ and PRF^+^ cells, after the first dose of vaccination — but mainly after the second dose (Figure 2A). In fact, the second dose of vaccination induced an increase in the cytotoxic function by CD4^+^ T cells in people < 60 years old but not in people > 60 years old (Figure 2A and Supplemental Figure 2C). Regarding CD8^+^, as it was observed in CD4^+^ T cells, aged people showed a lower SARS-CoV-2 S–specific CD8^+^ T cell response, based mainly on the production of IFN-γ and the cytotoxic capacity (CD107a^+^ and PRF^+^), 2 months after the second dose (Figure 2B).

To determine the quality of the specific T cell response to the SARS-CoV-2 vaccine, we analyzed the polyfunctionality of CD4^+^ and CD8^+^ T cells, which is defined by those cells that simultaneously produce multiple cytokines and degranulate (functions). In general, a low polyfunctional T cell response was observed after the vaccination with BNT162b2 mRNA vaccine in both aged and young participants (Figure 2C). However, a more polyfunctional memory CD4^+^ T cell response to SARS-CoV-2 was observed in people < 60 years old compared with people > 60 years old after the first dose, and a similar trend was observed after the second dose (Figure 2C). The polyfunctional profile of the rest of CD4^+^ T cell subsets showed a similar pattern, with the exception of TEMRA CD4^+^ T cells that presented higher polyfunctionality after the second dose in young people (Supplemental Figure 2D). To further characterize this SARS-CoV-2–specific T cell response to vaccination, we analyzed different combinations of the studied functions. CD4^+^ T cells expressing CD107a and PRF simultaneously were enriched in young people < 60 years old 2 months after the second dose of vaccination, confirming the higher T cell cytotoxic capacity of young people (Figure 2, C and D). Combinations including both IFN-γ and TNF-α and the ones including only IFN-γ were mainly observed in young people after the first and second dose of vaccination (Figure 2D). Furthermore, the percentages of T cells expressing 3 functions at the same time (e.g., IFN-γ^+^CD107a^+^PRF^+^ or IFN-γ^+^IL-2^+^TNF-α^+^) and other 2-function combinations (e.g., IFN-γ^+^IL-2^+^) were also higher in the < 60-year-old group than in > 60-year-old group (Supplemental Figure 2E). Therefore, the SARS-CoV-2 S–specific T cell response is lower and less polyfunctional in aged people after vaccination with the BNT162b2 mRNA vaccine.

### Lower thymic function and altered T cell homeostasis found in aged people are associated with a lower T cell response to the SARS-CoV-2 vaccine.

**Figure 3:**
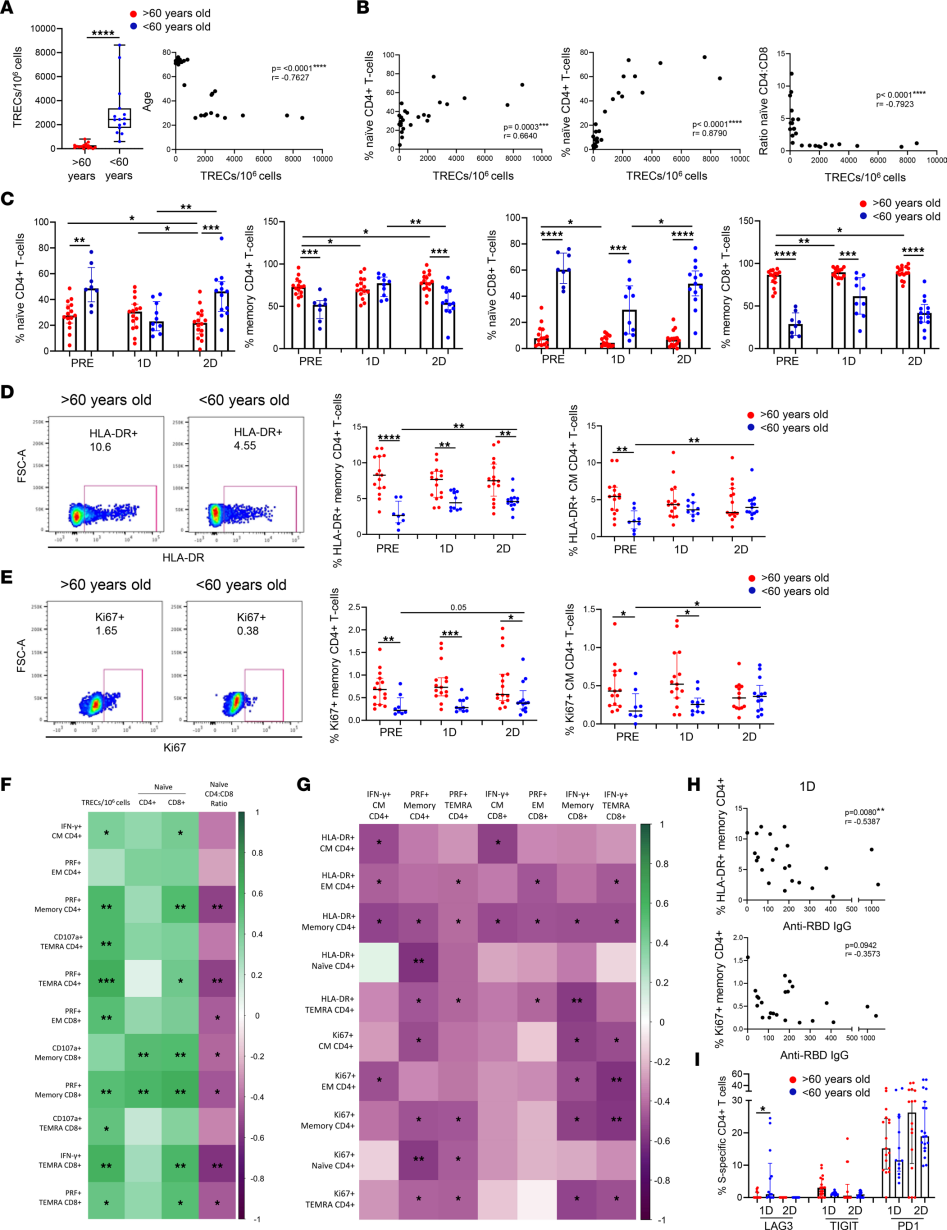
Lower thymic function and altered T cell homeostasis found in aged people are associated with a lower T cell response to the SARS-CoV-2 vaccine. (**A** and **B**) Bar graphs showing TREC/1 × 10^6^ cells as a measure of thymic function in > 60-year-old and < 60-year-old participants prior to vaccination (**A**, left) and the correlation of the TREC/1 × 10^6^ cells with age (**A**, right), naive CD4^+^ T cells (**B**, left), naive CD8^+^ T cells (**B**, middle), and naive CD4^+^/CD8^+^ T cell ratio (**B**, right). (**C**) Bar graphs representing the percentage of naive and memory CD4^+^ and CD8^+^ T cells in > 60-year-old (red) and < 60-year-old (blue) participants before SARS-CoV-2 vaccination (PRE), 3 weeks after the first dose (1D), and 2 months after the second dose (2D). (**D** and **E**) Dot plots representing the percentage of memory and CM CD4^+^ T cells expressing HLA-DR (**D**) and Ki67 (**E**) > 60-year-old (red) and < 60-year-old (blue) participants at the 3 time points (right). Pseudocolor dot plot graphs show representative data of memory CD4^+^ T cells from a > 60-year-old (red) and < 60-year-old (blue) donor expressing HLA-DR (**D**) and Ki67 (**E**) before vaccination (left). (**F** and **G**) Correlation matrixes representing associations of SARS-CoV-2 S–specific CD4^+^ and CD8^+^ T cells expressing IFN-γ or cytotoxicity markers 2 months after the second dose of vaccination with TREC/1 × 10^6^ cells and naive T cells (**F**) and with the percentage of HLA-DR^+^ and Ki67^+^ CD4^+^ T cells (**G**) before vaccination in all participants. (**H**) Correlation plots of anti–RBD IgG levels after the first dose of vaccination with the percentage of HLA-DR^+^ and Ki67^+^ CD4^+^ T cells before vaccination. (**I**) Bar graphs representing the percentage of SARS-CoV-2–specific CD4^+^ T cells expressing LAG-3 in > 60-year-old (red) and < 60-year-old (blue) participants at the 3 follow-up time points. Mann-Whitney *U* (**A**, **C**, **D**, **E**, and **I**), Wilcoxon (**A**, **C**, **D**, **E**, and **I**), and Spearman (**A**, **B**, **F**, **G**, and **H**) tests were used (*n =* 32). Friedman test was applied in **C** (Naive CD4^+^ T cells: > 60-year-old, *P =* 0.06, and < 60-year-old, *P =* 0.074; Memory CD4^+^ T cells: > 60-year-old, *P =* 0.071, and < 60-year-old, *P =* 0.074; Naive CD8^+^ T cells: > 60-year-old, *P =* 0.307, and < 60-year-old, *P =* 0.015; Memory CD8^+^ T cells: > 60-year-old, *P =* 0.035, and < 60-year-old, *P =* 0.091), **D** (Memory CD4^+^ T cells: > 60-year-old, *P =* 0.441, and < 60-year-old, *P =* 0.022; CM CD4^+^ T cells: > 60-year-old, *P =* 0.529, and < 60-year-old, *P =* 0.022), and **E** (Memory CD4^+^ T cells: > 60-year-old, *P =* 0.273, and < 60-year-old, *P =* 0.074; CM CD4^+^ T cells: > 60-year-old, *P =* 0.657, and < 60-year-old, *P =* 0.091). *P < 0.05, **P < 0.01, ***P < 0.001, ****P < 0.0001.

Once we demonstrated that aged people displayed a lower SARS-CoV-2–specific T cell response after vaccination, we investigated the immune defects that might be involved in the diminished response of this vulnerable population. In our group, we previously reported that thymic function failure and inflammation levels independently predict all-cause mortality in healthy elderly people (25). Thus, we studied if these factors could be associated with a lower SARS-CoV-2 vaccine response in aged participants. Thymic output can be measured through the presence of T cell receptor rearrangement excision circles (TREC) in naive T cells, which are indicators of recent thymic emigrants in humans (26). Thus, to determine the thymic function, we have adapted signal joint TREC (sjTREC) measurement with droplet digital PCR (ddPCR). Our results showed a considerably lower level of thymic function in people > 60 years old in comparison with participants < 60 years old; accordingly, there was a strong association between thymic function and age (Figure 3A). We observed a correlation between thymic function with naive CD4^+^ and CD8^+^ T cells and with the naive CD4^+^/CD8^+^ ratio (Figure 3B). In addition, decreased thymic function has been related to the phenomenon called memory inflation, which includes the alteration of the naive and memory T cell proportions in the periphery skewing toward memory T cells (27). This phenomenon was reflected in CD4^+^ and CD8^+^ T cell subset distribution in aged people > 60 years old, which displayed lower percentages of naive T cells and higher percentages of memory T cells than young participants (Figure 3C). Three weeks after the vaccination with the first dose, young participants < 60 years old showed a trend to decrease the percentages of naive T cells and to increase memory T cells and showed the restoration of T cell subset distribution that occurred 2 months after the second dose (Figure 3C). Thus, the differences in naive and memory T cells between aged and young people observed prior to vaccination were lost after the first dose but were restored 2 months after the second dose (Figure 3C).

According to lower thymic function and high memory inflation, elevated levels of T cell homeostatic proliferation and activation were found in the elderly people. Specifically, we observed that, prior to vaccination, people > 60 years old displayed a higher percentage of activated (HLA-DR^+^) and proliferating (Ki67^+^) memory and CM CD4^+^ T cells compared with people < 60 years old (Figure 3, D and E). After vaccination with 2 doses, an increase of HLA-DR^+^ and Ki67^+^ CD4^+^ T cells was observed in young participants, while older people showed a lack of further activation and proliferation through vaccination (Figure 3, D and E). Similar results were found in the rest of CD4^+^ T cell subsets (Supplemental Figure 3, A and B) and in some of the CD8^+^ T cell subsets (data not shown). Thymic function was negatively correlated with T cell activation in most of the subsets and was negatively correlated with proliferation in naive CD8^+^ T cells (Supplemental Figure 3C). Importantly, thymic dysfunction and the related defects in the homeostasis of a T cell compartment found in aged people prior to vaccination — expressed as higher T cell activation and proliferation and memory inflation — and memory inflation were correlated with a lower SARS-CoV-2–specific CD4^+^ and CD8^+^ T cell response after the 2-dose vaccination (Figure 3, F and G). Furthermore, the percentage of activated (HLA-DR^+^) memory CD4^+^ T cells was also inversely associated with anti–RBD IgG levels after the first dose, and the same trend was observed regarding proliferating (Ki67^+^) T cells (Figure 3H). Moreover, the vaccination altered the expression of immune checkpoints as LAG-3, PD-1, and TIGIT on CD4^+^ and CD8^+^ T cells being higher in the < 60-year-old group compared with the > 60-year-old group after the first or second dose in most of the T cell subsets (Supplemental Figure 3, D–F). The same result was observed regarding LAG-3 expression within SARS-CoV-2–specific CD4^+^ T cells, where this expression was higher in young participants after the first dose (Figure 3I). In contrast, PD-1 and TIGIT tended to be lower in the SARS-CoV-2–specific CD4^+^ T cells from < 60-year-old group compared with the > 60-year-old group (Figure 3I). In addition, virus-specific T cell response after vaccination was also inversely correlated with the expression of PD-1 in bulk CD4^+^ T cells before vaccination (Supplemental Figure 3G).

**Figure 4:**
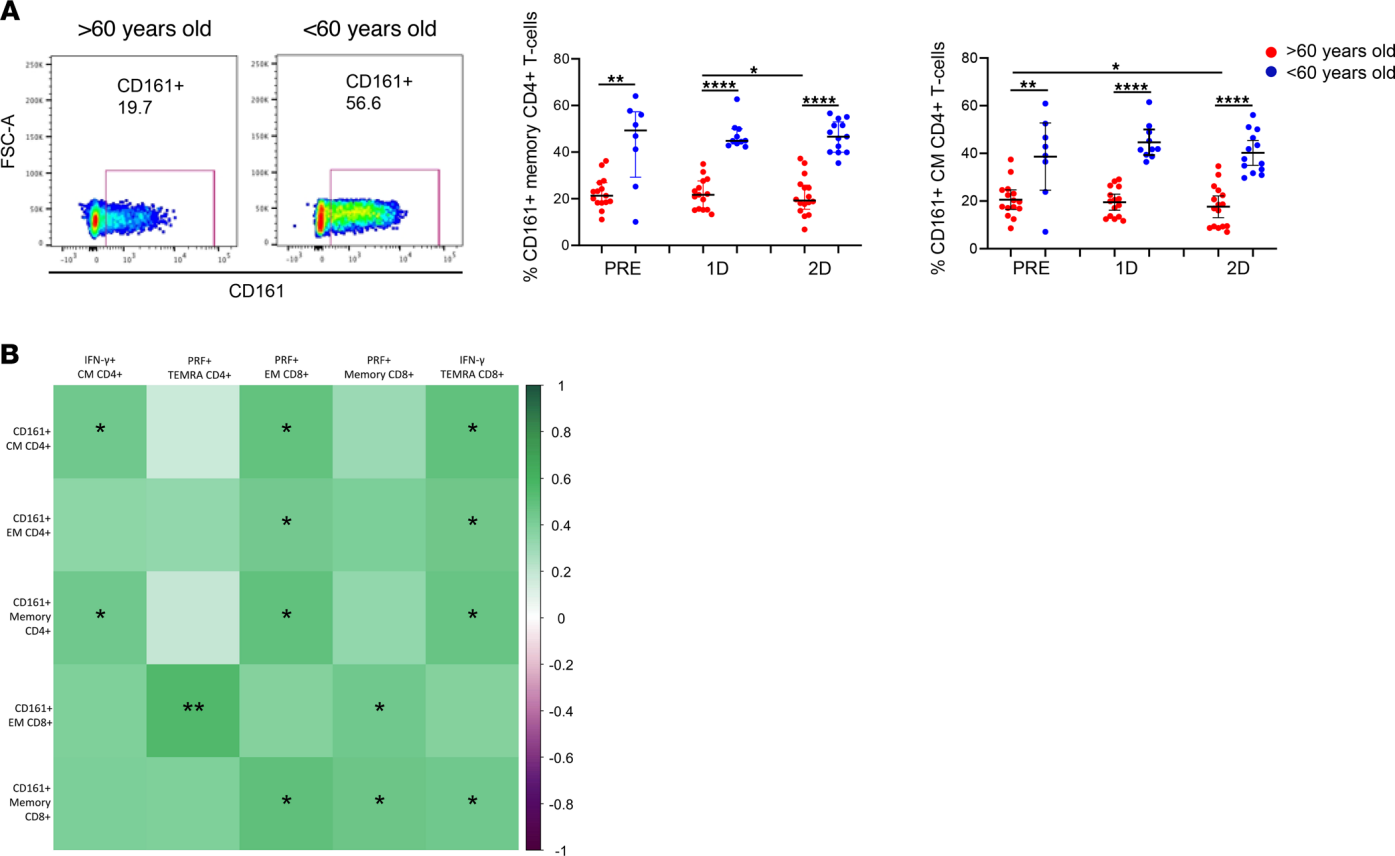
CD161-expressing T cell levels are associated with a higher SARS-CoV-2 vaccine T cell response. (**A**) Dot plots representing the percentage of memory and CM CD4^+^ T cells expressing CD161 in > 60-year-old (red) and < 60-year-old (blue) participants before SARS-CoV-2 vaccination (PRE), 3 weeks after the first dose (1D), and 2 months after the second dose (2D) (right). Pseudocolor dot plot graphs show representative data of memory CD4^+^ T cells from a > 60-year-old and < 60-year-old donor expressing CD161 before vaccination (left). (**B**) Correlation matrix representing associations of the percentage of CD161^+^ T cells before vaccination with SARS-CoV-2 S–specific CD4^+^ and CD8^+^ T cells expressing IFN-γ or cytotoxicity markers 2 months after the second dose of vaccination in all participants. Mann-Whitney *U* (**A**), Wilcoxon (**A**), and Spearman (**B**) tests were used (*n =* 32). Friedman test was applied in **A** (Memory CD4^+^ T cells: > 60-year-old, *P =* 0.091, and < 60-year-old, *P =* 0.368; CM CD4^+^ T cells: > 60-year-old, *P =* 0.159, and < 60-year-old, *P =* 0.549).

Other T cells that notably differed between aged and young participants were CD161^+^ T cells. These cells mainly produce IL-17 and are known to have an important contribution in pathogen clearance (28). CD161^+^ T cells presented higher levels in people < 60 years old compared with people > 60 years old, independently on the vaccination (Figure 4A). The percentage of CD161-expressing CD4^+^ and CD8^+^ T cells was positively associated with SARS-CoV-2 S–specific T cell response 2 months after the vaccination (Figure 4B).

### An impaired DC homing and functional capacity are associated with a lower T cell response to SARS-CoV-2 vaccine in aged people.

**Figure 5:**
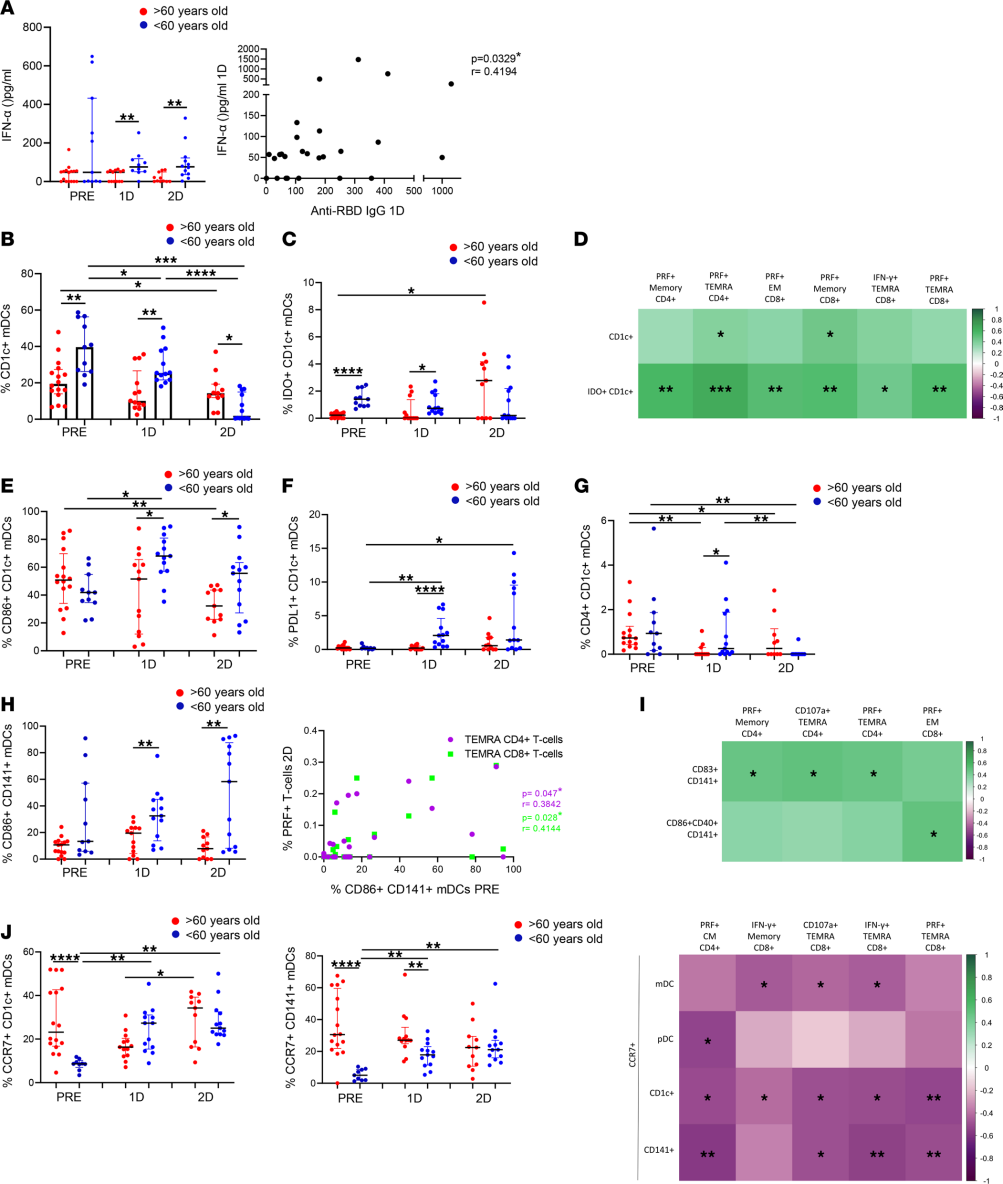
An impaired DC homing and functional capacity are associated with a lower T cell response to the SARS-CoV-2 vaccine in aged people. (**A**) Dot plots showing IFN-α production through CpG-A stimulation for 18 hours in > 60-year-old (red) and < 60-year-old (blue) participants before SARS-CoV-2 vaccination (PRE), 3 weeks after the first dose (1D), and 2 months after the second dose (2D) (left). Correlation analysis of IFN-α production with anti–RBD IgG levels 3 weeks after the first dose of vaccination (right). (**B** and **C**) Bar graphs representing the percentage of CD1c^+^ and IDO^+^CD1c^+^ mDCs in > 60-year-old (red) and < 60-year-old (blue) participants at the 3 follow-up time points. (**D**) Correlation matrix showing associations between the percentages of CD1c^+^ mDCs and IDO^+^CD1c^+^ mDCs before vaccination with SARS-CoV-2 S–specific T cells expressing cytokines or cytotoxicity markers 2 months after the second dose. (**E**–**G**) Dot plots showing the percentage of CD1c^+^ mDCs expressing CD86 (**E**), PD-L1 (**F**), and CD4 (**G**) in > 60-year-old (red) and < 60-year-old (blue) participants at the 3 time points. (**H**) Dot plots showing the percentage of CD141^+^ mDCs expressing CD86 (left) in > 60-year-old (red) and < 60-year-old (blue) participants at the 3 time points. Correlation plot between the percentage of CD86^+^CD141^+^ mDCs before vaccination and the percentage of S-specific PRF^+^ TEMRA CD4^+^ and CD8^+^ T cells 2 months after the second dose (right). (**I**) Correlation matrix showing associations between the percentage of CD141^+^ mDCs expressing activation markers after TLR-3 stimulation for 24 hours with SARS-CoV-2 S–specific CD4^+^ and CD8^+^ T cells expressing cytotoxicity markers. (**J**) Dot plots showing the percentage of CCR7^+^ mDCs in > 60-year-old (red) and < 60-year-old (blue) participants in the 3 follow-up time points (left and middle panels), and a correlation matrix representing associations of the percentage of mDCs expressing CCR7 with SARS-CoV-2 S–specific CD4^+^ and CD8^+^ T cells expressing cytokines or cytotoxicity markers 2 months after the second dose (right panel). Mann-Whitney *U* (**A**, **B**, **C**, **E**, **F**, **G**, **H**, and **J**), Wilcoxon (**A**, **B**, **C**, **E**, **F**, **G**, **H**, and **J**), and Spearman (**A**, **D**, **H**, **I**, and **J**) tests were used (*n =* 32). Friedman test was applied in **A** (> 60-year-old, *P =* 0.801; < 60-year-old, *P =* 0.717), **B** (> 60-year-old, *P =* 0.169; < 60-year-old, *P =* <0.0001), **C** (> 60-year-old, *P =* 0.147; < 60-year-old, *P =* 0.027), **E** (> 60-year-old, *P =* 0.018; < 60-year-old, *P =* 0.086), **F** (> 60-year-old, *P =* 0.381; < 60-year-old, *P =* 0.013), **G** (> 60-year-old, *P =* 0.042; < 60-year-old, *P =* 0.034), **H** (> 60-year-old, *P =* 0.223; < 60-year-old, *P =* 0.234), and **J** (CD1c mDCs: > 60-year-old, *P =* 0.121; < 60-year-old, *P =* 0.001 and CD141 mDCs: > 60-year-old, *P =* 0.459; < 60-year-old, *P =* 0.001).

In addition to the alteration of the adaptive immunity associated with lower response to the vaccine in the elderly, there is a remodeling of the innate immune system with aging (29). Thus, we next studied plasmacytoid DCs (pDCs) and myeloid DCs (mDCs) (Supplemental Figure 4A), innate immune cells with a key role in the modulation of T cell response (30). We first observed a decrease in pDC percentages 2 months after the second dose (Supplemental Figure 4B). Although we did not find differences between people > 60 and < 60 years old in pDC levels (Supplemental Figure 4B), we observed a considerable difference in pDC functional capacity (Figure 5A). When cells were stimulated in a TLR-9–dependent manner by CpG-A, a lower IFN-α production was observed in aged people compared with young participants, both after the first and second dose of the SARS-CoV-2 vaccine (Figure 5A, left panel). Interestingly, we also observed how this functional capacity of the pDCs was associated with anti–RBD IgG levels after the first dose (Figure 5A, right panel).

Next, we focused on mDC subsets, including CD1c^+^, CD16^+^, and CD141^+^ mDCs (Supplemental Figure 4A). Our results showed a higher percentage of CD1c^+^ mDCs in < 60-year-old than in > 60-year-old people prior to vaccination and after the first dose (Figure 5B), a mDC subpopulation that modulates CD4^+^ T cell response (30). In contrast, a notable decrease in CD1c^+^ mDC percentage was observed only in young people 2 months after the second dose of the SARS-CoV-2 vaccine (Figure 5B). The same result was found regarding the percentage of integrin-β7–expressing CD1c^+^ mDCs (Supplemental Figure 4C, left panel), with integrin-β7 as a maker of cell homing to gut. It is remarkable, that the decrease in integrin-β7^+^CD1c^+^ mDCs was correlated with a higher IFN-γ production by TEMRA CD4^+^ and CD8^+^ T cells in response to SARS-CoV-2 two months after vaccination (Supplemental Figure 4C, right panel). Furthermore, higher expression of indoleamine 2,3-dioxygenase (IDO) was found on CD1c^+^ mDCs from young people < 60 years old in most of the studied time points (Figure 5C), and the percentage of CD1c^+^ and IDO^+^CD1c^+^ mDCs prior to vaccination were correlated with SARS-CoV-2–specific T cell response 2 months after the second dose (Figure 5D). Interestingly, although no significant correlation was found, a trend was observed between IDO expression on CD1c^+^ and CD16^+^ mDCs and proliferating (Ki67^+^) memory CD4^+^ T cells (Supplemental Figure 4D). The expression of CD86, PD-L1, and CD4 on CD1c^+^ mDCs, markers related to the modulation of T cell response, was also higher in young people (Figure 5, E–G).

It is also known that CD141^+^ mDCs are involved in the regulation of CD8^+^ T cell response (30). In this study, higher CD141^+^ mDC levels were found in the < 60-year-old group after the first dose, compared with > 60-year-old group (Supplemental Figure 4E). As occurred with CD1c^+^ mDCs, young people also displayed a higher expression of the costimulatory molecule CD86 (Figure 5H, left panel) after vaccination in this subset, and this was correlated with SARS-CoV-2–specific cytotoxic response (PRF^+^) by TEMRA T cells (Figure 5H, right panel). In order to study the functional capacity of mDCs, cells were stimulated with Poly(I:C), an agonist of TLR-3. In all participants, the percentages of activated CD141^+^ mDCs (CD86^+^CD40^+^ and CD83^+^) were increased following TLR-3 stimulation, before vaccination and after the first dose (Supplemental Figure 4F). Nevertheless, 2 months after the second dose, CD141^+^ mDCs were not successfully stimulated via TLR-3, since they were already activated by the vaccination (Supplemental Figure 4E). This effect was also observed in the rest of mDC subsets (Supplemental Figure 4, G and H). Even though no differences were found between aged and young people in CD141^+^ mDC response, a higher functional capacity of CD141^+^ mDCs prior to vaccination was positively associated with SARS-CoV-2–specific T cell response after vaccination (Figure 5I). Additionally, other DC markers were altered after the vaccination on different subsets. CCR7, a chemokine receptor that orchestrates DC migration to draining lymph nodes (31), was highly expressed in aged people > 60 years old before vaccination, and its expression was increased after the first and second dose mostly in young people, with no response in elderly people (Figure 5J, left and middle panels). A higher CCR7 expression on DCs prior to vaccination was associated with a lower SARS-CoV-2–specific T cell response (Figure 5J, right panel).

**Figure 6:**
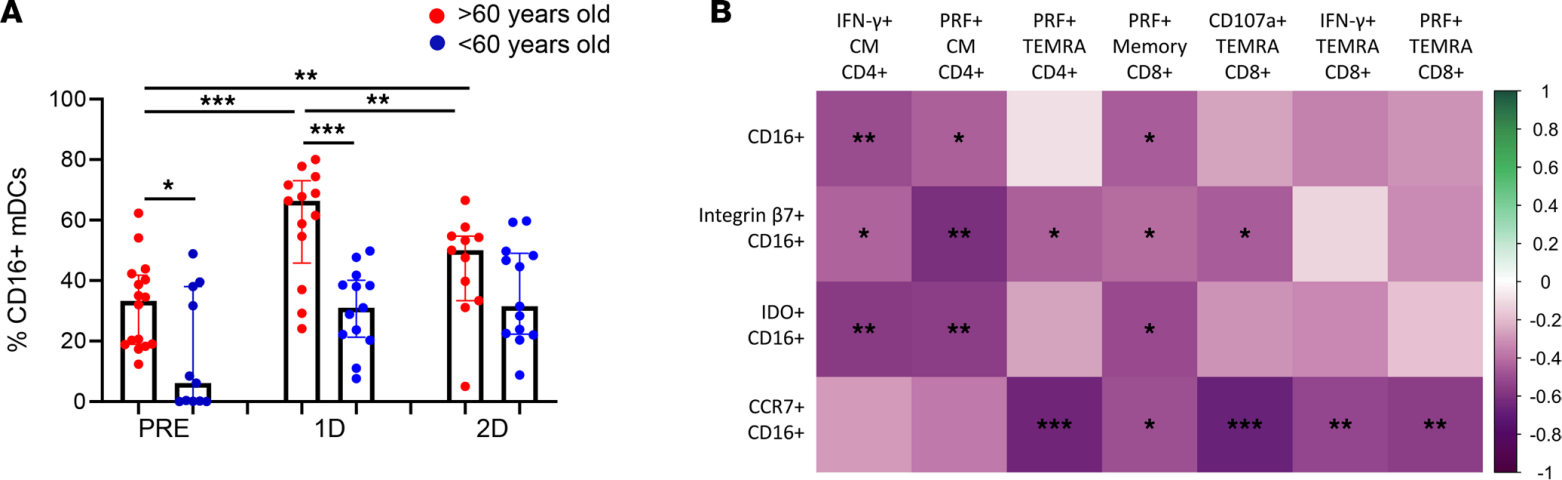
Higher CD16^+^ myeloid DC percentage from aged people is associated with a lower T cell response to the SARS-CoV-2 vaccine. (**A**) Bar graphs representing the percentage of CD16^+^ mDCs in > 60-year-old (red) and < 60-year-old (blue) participants before SARS-CoV-2 vaccination (PRE), 3 weeks after the first dose (1D), and 2 months after the second dose (2D) of vaccination. (**B**) Correlation matrix showing associations between the percentage of CD16^+^ mDCs and CD16^+^ mDCs expressing integrin-β7 and IDO with SARS-CoV-2 S–specific CD4^+^ and CD8^+^ T cells expressing cytotoxicity markers. Mann-Whitney *U* (**A**), Wilcoxon (**A**), and Spearman (**B**) tests were used (*n =* 32). Friedman test was applied in **A**, left (> 60-year-old, *P =* 0.01; < 60-year-old, *P =* 0.148).

Due to their role in the modulation of inflammatory responses, CD16^+^ mDCs were also studied (32). In general, people > 60 years old displayed higher percentages of CD16^+^ mDCs than participants < 60 years old (Figure 6A). Focusing on the effect of SARS-CoV-2 vaccination, an increase in CD16^+^ mDC percentages was observed mainly after the first dose but also after the second dose only in aged participants (Figure 6A). The percentage of CD16^+^ mDCs and the percentages of CD16^+^ mDCs expressing integrin-β7, IDO, and CCR7 were inversely associated with S-specific T cell response (Figure 6B). Hence, our results indicate that the higher levels of CD16^+^ mDCs, which have a proinflammatory function, along with the impaired DC homing and functional capacity found in aged people were associated with a lower T cell response to SARS-CoV-2 vaccine.

### Higher monocyte-mediated proinflammatory profile found in aged people is associated with a lower T cell response to SARS-CoV-2 vaccine.

**Figure 7:**
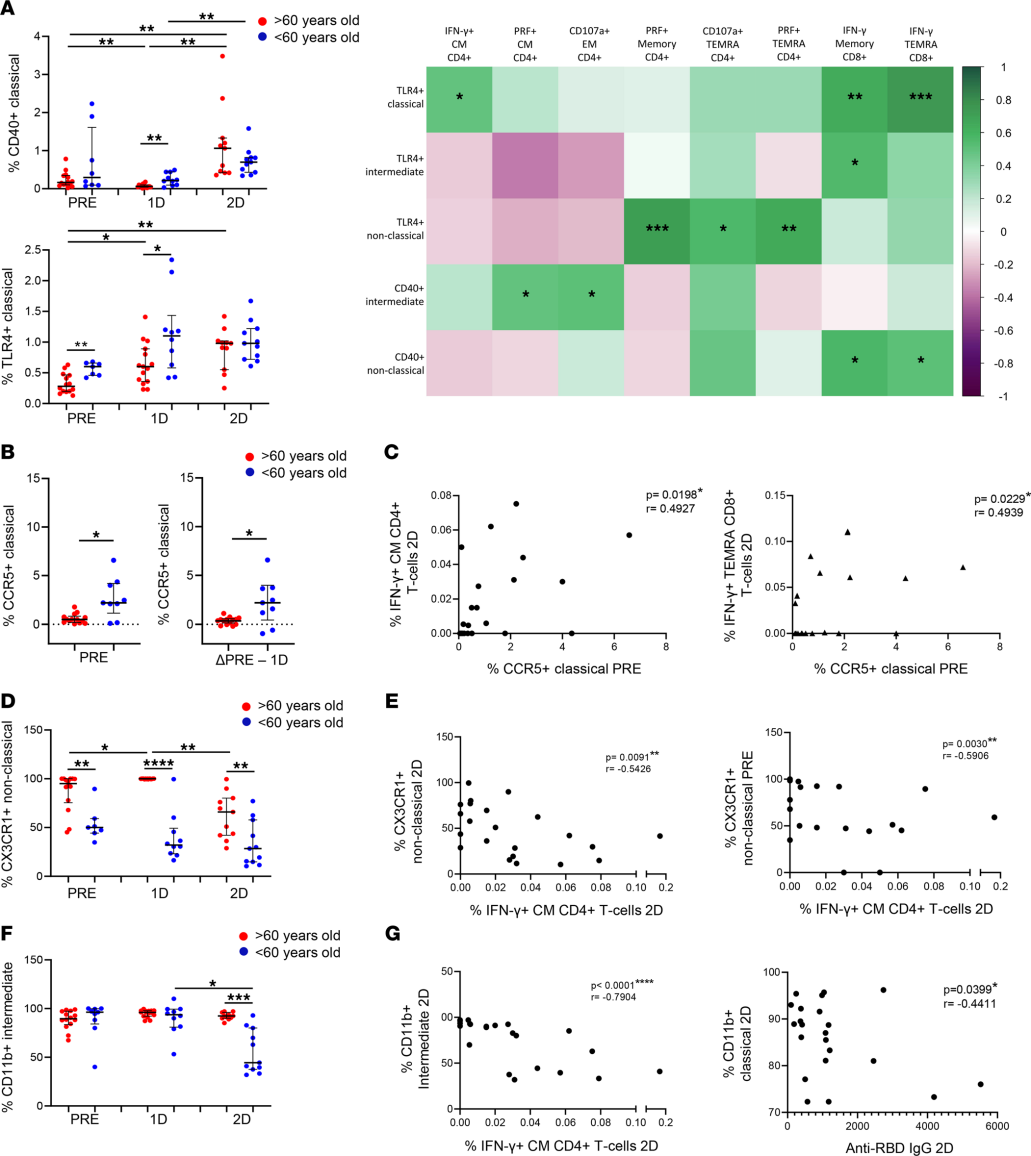
Diminished monocyte activation and homing found in aged people are related to a lower SARS-CoV-2 vaccine response. (**A**) Dot plots representing the percentage of classical monocytes expressing CD40 (top) and TLR-4 (bottom) in > 60-year-old (red) and < 60-year-old (blue) participants before SARS-CoV-2 vaccination (PRE), 3 weeks after the first dose (1D), and 2 months after the second dose (2D) of vaccination (left). A correlation matrix representing the percentage of monocytes expressing CD40 and TLR-4 prior to vaccination with SARS-CoV-2 S–specific CD4^+^ and CD8^+^ T cells expressing cytokines or cytotoxicity markers 2 months after the second dose (right). (**B** and **C**) Dot plots representing the percentage of classical monocytes expressing CCR5 before vaccination (**B**, left), the fold of decrease in the percentage of CCR5^+^ cells after the first dose (**B**, right), and correlation analysis of the percentage of CCR5^+^ classical monocytes prior to vaccination with SARS-CoV-2 S–specific CD4^+^ and CD8^+^ T cells expressing IFN-γ 2 months after the second dose (**C**). (**D** and **E**) Dot plots showing the percentages of nonclassical monocytes expressing CX3CR1 in > 60-year-old (red) and < 60-year-old (blue) participants at the 3 follow-up time points (**D**). Correlations of the percentage of nonclassical monocytes expressing CX3CR1 2 months after the second dose (**E**, left) and prior vaccination (**E**, right) with SARS-CoV-2 S–specific IFN-γ^+^ CD4^+^ T cells after the second dose. (**F** and **G**) Dot plots showing the percentages of monocytes expressing CD11b in > 60-year-old (red) and < 60-year-old (blue) participants at the 3 follow-up time points (F). Correlation plots between the percentage of CD11b^+^ classical monocytes with SARS-CoV-2 S–specific IFN-γ^+^ CD4^+^ T cells (**G**, left) and anti–RBD IgG levels (**G**, right) 2 months after the second dose. Mann-Whitney *U* (**A**, **B**, **D**, and **F**), Wilcoxon (**A**, **B**, **D**, and **F**), and Spearman (**A**, **C**, **E**, and **G**) tests were used (*n =* 28). Friedman test was applied in **A** (CD40: > 60-year-old, *P =* 0.006, and < 60-year-old, *P =* 0.050; TLR4: > 60-year-old, *P =* 0.042, and < 60-year-old, *P =* 0.779), **D** (> 60-year-old, *P =* 0.002; < 60-year-old, *P =* 0.717) and **F** (> 60-year-old, *P =* 0.607; < 60-year-old, *P =* 0.368).

As one of the main players in inflammatory responses and the inflammaging phenomenon (29, 33), monocytes were analyzed in this study. Specifically, previously studied activation and homing markers (34, 35) were analyzed in classical (CD14^++^CD16^–^), intermediate (CD14^++^CD16^+^) and nonclassical monocytes (CD14^+^CD16^++^) (Supplemental Figure 5A). Our results show that, in all participants, SARS-CoV-2 vaccination induced an increase in the expression of activation markers and TLR in classical and intermediate monocytes, including CD40, TLR-4 (Figure 7A, left panel, and Supplemental Figure 5B), TLR-2, and CD49d (Supplemental Figure 5, C and D). Nevertheless, the monocyte activation levels after the first dose were higher in people < 60 years old than people > 60 years old (Figure 7A and Supplemental Figure 5B). The expression levels of CD40 and TLR-4 before vaccination were positively correlated with SARS-CoV-2–specific T cell response 2 months after the second dose (Figure 7A, right panel). The expression of CCR5, a monocyte chemokine receptor, is modulated after activation (36). Our results show that CCR5 expression before vaccination was higher in monocytes from young people compared with those from aged people (Figure 7B, left panel). However, CCR5 expression was downregulated after the first dose of SARS-CoV-2 vaccination, with this decrease being less pronounced in aged people (Figure 7B, right panel). Importantly, basal CCR5 expression was also directly correlated to IFN-γ production by CM CD4^+^ and TEMRA CD8^+^ T cells after SARS-CoV-2 vaccination (Figure 7C). Regarding other monocyte chemokine receptors, 2-dose vaccination induced a decrease in the percentage of intermediate monocytes expressing CCR2 and CD11b and nonclassical monocytes expressing CX3CR1; these percentages were lower in people < 60 years old than in people > 60 years old (Figure 7, C and D, and Supplemental Figure 5E). The lower percentages of monocytes expressing these chemokine receptors 2 months after the second dose of vaccination were inversely correlated with SARS-CoV-2 T cell response, and CD11b was also negatively correlated with anti–RBD IgG levels (Figure 7, E–G). Furthermore, the expression of CX3CR1 in nonclassical monocytes prior to vaccination was also inversely associated with the SARS-CoV-2–specific T cell response (Figure 7F). Moreover, monocyte tissue factor (CD142) expression is known to be induced by several inflammatory stimuli (37, 38). Here, we found an increase in the expression of tissue factor on classical monocytes after SARS-CoV-2 vaccination in both groups (Supplemental Figure 5F, left panel). In addition, tissue factor expression levels were higher on intermediate monocytes from young participants after the first dose (Supplemental Figure 5F, right panel).

**Figure 8:**
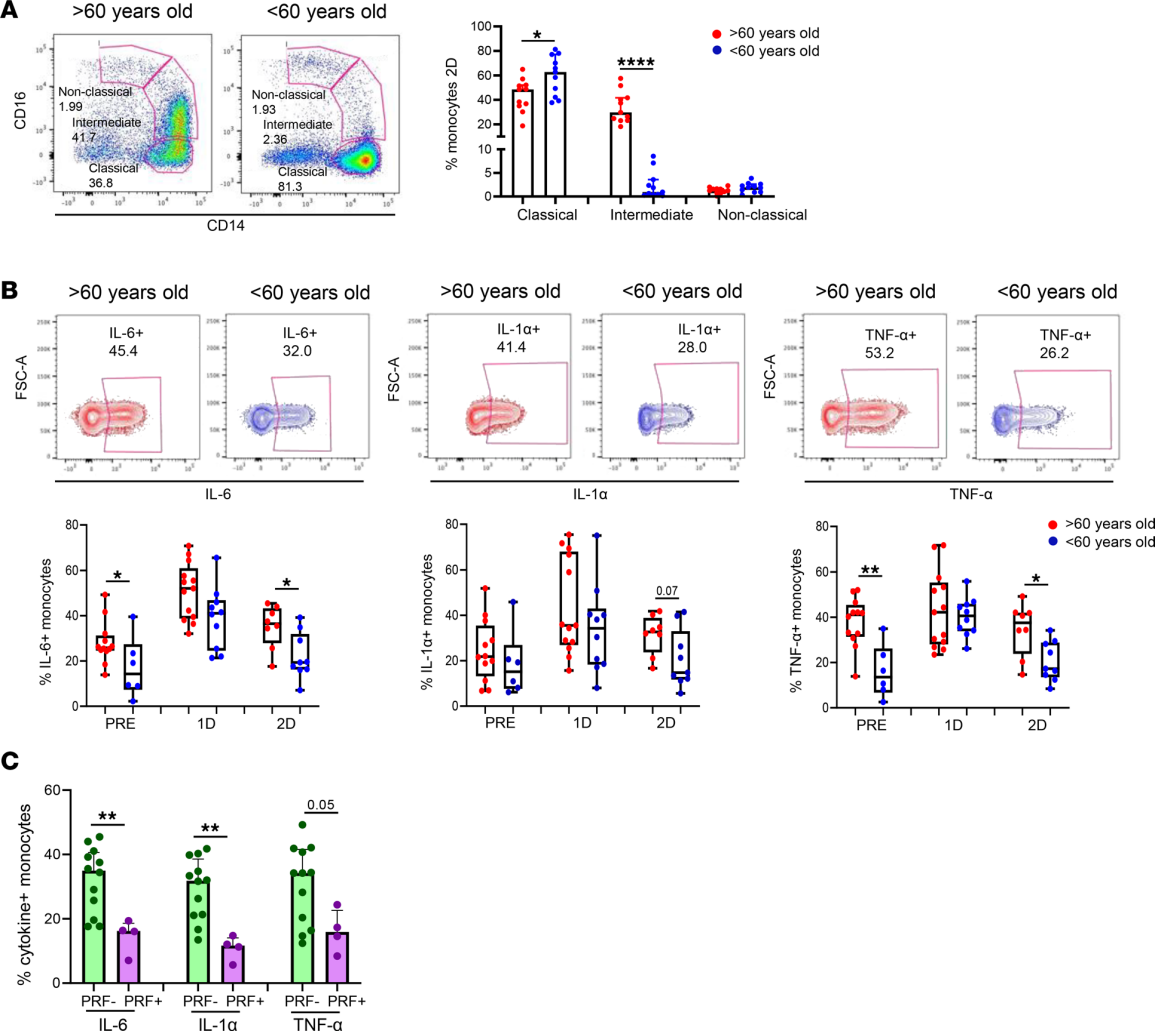
Higher monocyte-mediated proinflammatory profile found in aged people is associated with a lower T cell response to SARS-CoV-2 vaccine. (**A**) Bar graphs representing the percentage of classical, intermediate, and nonclassical monocytes in > 60-year-old (red) and < 60-year-old (blue) participants before SARS-CoV-2 vaccination (PRE), 3 weeks after the first dose (1D), and 2 months after the second dose (2D) of vaccination (right). Pseudocolor plots showing representative data of a > 60-year-old and < 60-year-old participant 2 months after the second dose (left). (**B**) Box-and-whisker plots (min to max) representing the percentage of IL-6^+^, IL-1α^+^, and TNF-α^+^ monocytes upon TLR-4 stimulation in > 60-year-old (red) and < 60-year-old (blue) participants at the 3 time points (bottom). Contour plots showing representative data of the percentage of cytokine^+^ monocytes from a > 60-year-old and < 60-year-old subject 2 months after the second dose (top). (**C**) Bar graphs showing the percentage of cytokine (IL-6, IL1-α, and TNF-α) producing monocytes on individuals with a cytotoxic SARS-CoV-2 S–specific T cell response and the ones with a negative response. The percentage of specific PRF^+^ T cells higher than 0.01 was considered as a positive cytotoxic T cell response. Mann-Whitney *U* and Wilcoxon tests were used (*n =* 26). Friedman test was applied in **B**, (IL-6: > 60-year-old, *P =* 0.223, and < 60-year-old, *P =* 0.368; IL-1α: > 60-year-old, *P =* 1.00, and < 60-year-old, *P =* 0.368; TNF-α: > 60-year-old, *P =* 0.223, and < 60-year-old, *P =* 0.368).

It has been previously reported that intermediate monocytes are expanded in the blood of patients with systemic infections (39). Here, we found a considerable increase in the percentage of intermediate monocytes, along with a decrease in classical monocytes, after the 2-dose SARS-CoV-2 vaccination only in people > 60 years old (Figure 8A). No differences were found prior to vaccination or after the first dose (Supplemental Figure 5G). Intermediate monocytes are known to secrete TNF-α, IL-1β, IL-6, and CCL3 upon TLR stimulation (39). In this study, we stimulated cells in a TLR-4–dependent manner by adding LPS. Importantly, vaccinated aged people showed a higher production of IL-6, IL-1α, and TNF-α by monocytes upon LPS stimulation compared with young participants, mainly after the second dose (Figure 8B). Lastly, we discovered that participants who did not show a cytotoxic SARS-CoV-2–specific T cell response (PRF^–^) produced higher levels of inflammatory cytokines by monocytes after vaccination compared with the ones presenting cytotoxic T cells (PRF^+^) after SARS-CoV-2 vaccination (Figure 8C). In summary, although monocytes from aged people showed lower levels of activation and homing after vaccination, they produced higher levels of proinflammatory cytokines upon LPS stimulation, and this was inversely associated with SARS-CoV-2–specific T cell response.

## Discussion

Aging is associated with impaired COVID-19 vaccine response (20). We confirmed and extended that the age-related immunological defects were characterized mostly by a lower magnitude and polyfunctionality of SARS-CoV-2–specific T cell response. The adaptive and innate immune factors behind these defects in aged people included an alteration in T cell homeostasis parameters fueled by lower thymic function and higher T cell activation and proliferation, DC dysfunction, and a higher proinflammatory profile in circulating monocytes.

In spite of the efficacy of BNT162b2 mRNA vaccine to prevent severe COVID-19 outcomes, vulnerable populations such as elderly people remain at risk. Several studies have reported lower humoral response in aged participants following vaccination (19–23). In accordance to these findings, we observed an inverse association between anti–RBD IgG levels with age at 2 months after vaccination. Moreover, we also found a diminished specific T cell response to SARS-CoV-2 vaccine in aged people, confirming previous results (20, 21). In addition to the magnitude, high quality of SARS-CoV-2–specific T cell function is required to achieve an effective vaccine response, which has not been completely determined yet. We found that specific T cells, in addition to producing different cytokines, exhibited a cytotoxic response to the vaccination, which is diminished in the elderly. Although young people showed a higher polyfunctional response than aged people, the BNT162b2 mRNA vaccine did not induce a high polyfunctional T cell immunity in general. This could be one of the key factors that might explain the absence of vaccine efficacy to avoid new infections over time, independent of the virus variant of concern (VOC).

Age-related changes in the immune system, known as immunosenescence, cause a subclinical immune deficiency that involves a reduced antiviral function and vaccine response (5). One of the most known changes of the immune aging is the involution of the thymus (40, 27). In our study, aged people exhibited thymic dysfunction and the subsequent memory inflation, reproducing previous findings (41). Remarkably, it has been described that thymic function failure predicts all-cause mortality in healthy aged people (25) and has a relevant role in viral infections such as HIV-1 infection (26, 42, 43). Moreover, it has been suggested that thymic aging might have an important implication in COVID-19 disease severity (44, 45). Importantly, we discovered that the thymic dysfunction, along with the memory inflation and a higher homeostatic T cell proliferation and activation found in aged people, correlated with a lower response to SARS-CoV-2 vaccination. Accordingly, a lower activity of the thymus has been previously associated with diminished responsiveness to vaccination against other viruses, such as yellow fever virus (46). In addition, thymic dysfunction was associated with a higher homeostatic T cell proliferation and activation. In fact, the age-dependent shift in the T cell population from naive to memory phenotype induces homeostatic T cell proliferation to compensate for the diminished T cell thymic output (47, 48). The higher T cell activation status found in aged people prior vaccination might be the reason why there is a lack of further activation of T cells after SARS-CoV-2 vaccination in this population. This was reflected in a lower increase in the expression of T cell activation markers, such as HLA-DR, in aged people after vaccination, as well as lower expression of checkpoint receptors as LAG-3 in both bulk and SARS-CoV-2–specific CD4^+^ T cells, a marker that is overexpressed after cellular activation (49).

One of the most remarkable findings of our study is the tight association of CD161 expression levels with age. CD161 is a C-type lectin receptor that is expressed in both T and NK cells (50). CD161^+^ T cells has been associated with IL-17 production and, hence, with pathogen clearance (51). In fact, we have previously observed that CD161^+^CD8^+^ T cells were related with HIV and hepatitis C virus–specific (HCV-specific) T cell polyfunctionality, which is essential for HIV spontaneous control and HCV spontaneous clearance (28). Results presented herein suggest the absence of a CD161 marker as a hallmark of T cell immunosenescence in accordance with recent findings, which show an inverse correlation of CD161 expression on CD8^+^ T cells with age, independent of CMV infection (52). Importantly, a reduction in the frequency of CD161^+^CD8^+^ T cells was found in peripheral blood of severe COVID-19 patients (53). In this study, we found that CD161 expression on T cells was tightly associated with SARS-CoV-2 vaccine response, highlighting this molecule as a potential target for immunotherapeutic strategies for age-related disease therapies and vaccine response in the elderly.

The regulation of the immune response highly depends on the function of DCs (30); however, their role in immune aging needs to be better understood. In this work, we found an altered DC subset distribution and an impaired DC homing and function with aging. The levels of CD1c^+^ mDCs, cells responsible for the modulation of CD4^+^ T cell response, were lower in aged people prior to vaccination, and this was related to a lower SARS-CoV-2 T cell response after vaccination. Accordingly, Schulz et al. previously observed an association between DC numbers and the response to yellow fever vaccine (46). Furthermore, the lower numbers of CD1c^+^ mDCs and integrin-β7–expressing CD1c^+^ mDCs observed after the second dose indicates that vaccination may induce a CD1c^+^ mDC migration to gut and probably to other tissues. A decrease in peripheral blood CD1c^+^ mDC numbers has been found in patients with severe COVID-19, due to a migration of these cells from blood to the lungs (54, 55). Interestingly, the vaccine-induced CD1c^+^ homing was barely observed in aged people, and this was also associated with a decreased SARS-CoV-2–specific T cell response, suggesting that aging might affect the capacity of CD1c^+^ mDCs to migrate and might consequently affect their ability to modulate T cell function. A lower expression of receptors such as CD86 in aged people could be indicative of a less efficient CD1c^+^ mDC activation and T cell costimulation after vaccination.

In addition to homing, DC function is also altered with aging. In response to TLR-3, TLR-7/8, and TLR-9 ligand stimulation, CD141^+^ mDCs and pDCs from aged participants secreted lower levels of IL-6, IL-12, and TNF-α (56). Other studies have reported that, in response to influenza A virus infection and West Nile infection, pDCs from older adults produced less type I IFN (57). Accordingly, our results show lower TLR-9–dependent IFN-α production by pDCs in aged people, both before and after the vaccination, against SARS-CoV-2. According to this, patients with COVID-19 presented lower TLR-9–mediated IFN-α production than healthy donors (54). This might be of great importance, since type I IFNs control the innate and adaptive immune system and induce cells’ antiviral state via the upregulation of IFN-stimulated genes that inhibit the replication and spread of viruses (58). Remarkably, we found that CD141^+^ mDCs may also have an important implication in SARS-CoV-2 vaccination immunity, which is one of the cell types that is known to be depleted in COVID-19 patients and which is important for disease progression (55, 59). Here, we discovered that TLR-3–mediated CD141^+^ mDC activation capacity was directly associated with SARS-CoV-2 T cell response following vaccination. This is in accordance with the important function of CD141^+^ mDCs in antigen presentation to T cells (60, 61).

Other important innate immune cells that might have relevant roles in vaccine response are monocytes. In this study, we reported that SARS-CoV-2 vaccination caused monocyte activation and homing, reflected by higher expression of activation markers (CD40, TLR-4, TLR-2, and CD49d) and lower percentages of CCR2-, CD11b-, and CX3CR1-expressing intermediate and nonclassical monocytes after vaccination. It has been described that, in addition to DCs, these monocyte subsets also migrate from bloodstream to lungs in patients with COVID-19 (55). However, vaccine-induced monocyte homing was found mainly in young people. As we observed in CD1c^+^ mDCs, less monocyte homing was associated with a lower specific T cell response to vaccination. Although monocytes do not have a main role in the modulation of T cell response, monocytes are known to have the ability to prime tissue-resident T cells via cytokine production (62). Thus, a deficit in monocyte migration to inflammatory sites might also negatively affect T cell response.

One of the key age-related immune defects is the phenomenon called inflammaging, which is a persistent increase in basal proinflammatory phenotype found in the elderly (4). Monocytes are one of the principal players of inflammaging (29, 33). Inflammation is a critical factor in COVID-19 progression, where a monocyte-driven cytokine storm induces a hyperinflammatory phenotype, leading to a more severe symptomatology in COVID-19 patients (63). Here, we discovered higher TLR-4–mediated proinflammatory cytokine production by monocytes from aged people after SARS-CoV-2 vaccination; importantly, this cytokine production was inversely correlated with specific T cell response. This is in accordance with previously published studies, describing the role of inflammatory monocytes in the suppression of vaccine responses (64, 65). In fact, increased gene expression of inflammatory responses and TNF-α signaling via NF-κB were reported after COVID-19 vaccination (66). Importantly, plasma TNF-α levels in aged people were associated with a poorer antibody response following SARS-CoV-2 vaccination (21). According to our results from monocytes, aged people showed increased numbers of CD16^+^ mDCs, a DC subset that also participates in inflammatory responses (32). These higher CD16^+^ mDC levels were inversely associated with SARS-CoV-2–specific T cell response after vaccination. Altogether, a higher capacity of monocytes to produce proinflammatory cytokines, increased CD16^+^ mDC numbers, and even the higher T cell activation status found in aged people are probably associated with the inflammaging phenomenon, which — at the same time — is related to lower vaccine immunity.

In the present study, numerous age-related immune deficits associated with a lower SARS-CoV-2 vaccination response were described. It may be interesting to carry out the same determinations long-term after SARS-CoV-2 vaccination, to study how these immune alterations might be contributing to a less-durable protection after COVID-19 vaccination found in aged people. Nevertheless, the described age-associated immune defects are already observed 2 months after the second dose; therefore, these defects are expected to be maintained or even increase with a longer follow-up and might explain why waning in mRNA vaccine effectiveness occurs at a greater rate among older people. Another limitation of the study might be the sample size. However, we performed an exhaustive and comprehensive analysis of age-associated immune deficits, and the differences and associations among the contrasts were clear. Moreover, functional assays were performed with peripheral blood mononuclear cells (PBMCs), instead of isolated cells; therefore, some of the alterations found in the studied cells’ responses might be influenced by bystander cells. However, the analysis of functional capacity using PBMCs reflects better what is occurring in physiological conditions. Lastly, another limitation of this work could be that we only studied the response to BNT162b2 mRNA vaccine. Nevertheless, it is one of the most administered COVID-19 vaccines, and it would not be surprising that other vaccines present similar results, especially other mRNA vaccines.

In conclusion, we describe age-related innate and adaptive immune deficits associated with a lower SARS-CoV-2 vaccination response. Based on the results from this study, we suggest that the putative causal drivers of the lower vaccine response in aged people include: (a) thymic dysfunction, which induces a memory inflation (less naive T cells and higher memory T cells) and directly influences T cell response to the vaccine in aged people; (b) defective DC migration and activation, which cause a lower DC-mediated T cell costimulation and, therefore, a lower T cell response to the vaccine; and (c) the inflammaging, induced by a higher production of proinflammatory cytokines by monocytes and accompanied by a higher activation of the immune system, which causes an inefficient further activation of the immune cells in response to new antigens or, in this case, to the vaccine. These findings contribute to a better understanding of why aged people are less capable of responding to SARS-CoV-2 vaccination and might be relevant for the improvement of the current vaccination strategies — especially in this vulnerable population — and for the development of more efficient prototypes for the general population.

## Methods

### Study participants.

Fifty-four participants vaccinated with the BNT162b2 mRNA vaccine against SARS-CoV-2 were included in this study. Participants were stratified by age: participants < 60 years old were categorized as young (*n =* 33) and those > 60 years old categorized as aged (*n =* 21); the median ages of young and aged participants were 29 (IQR 26–49) and 73 (IQR 72–74), respectively. Young participants were workers from the IBiS, and aged participants were community volunteers from Seville, Dos Hermanas (both in Seville, Spain), and Rota (Cadiz, Spain). Inclusion criteria included participants with self-sufficient health status, and participants were excluded if they had a diagnosis of dementia, had active infections, or were admitted to a hospital during the last 6 months. Three young participants were excluded from the study due to a positive result for SARS-CoV-2 PCR or SARS-CoV-2 RBD–specific antibodies prior to vaccinaton. Peripheral blood samples were extracted from February to November 2021.

### Cell and plasma isolation.

PBMCs and plasma were isolated from study participants’ blood. PBMCs were isolated using BD Vacutainer CPT Mononuclear Cell Preparation Tubes (with sodium heparin, BD Biosciences) in a density gradient centrifugation at the same day of blood collection. CPTs were centrifuged at 1,811*g* for 20 minutes at room temperature (RT). Afterward, PBMCs were cryopreserved in freezing medium (90% of FBS [Thermo Fisher Scientific] + 10% dimethyl sulfoxide [DMSO; PanReac AppliChem]) in liquid nitrogen until further use. Plasma samples were obtained using BD Vacutainer PET EDTA Tube centrifugation at 1,811*g* for 5 minutes at room temperature, were aliquoted, and were cryopreserved at –80°C until further use.

### Cell stimulation.

PBMCs were thawed, washed using RPMI 1640 (Thermo Fisher Scientific), and rested for 1 hour in 0.25 μL/mL DNase I (Roche Diagnostics) containing R-10 complete medium (RPMI 1640 supplemented with 10% FBS, 100 U/mL penicillin G, 100 L/mL streptomycin sulfate [Thermo Fisher Scientific], and 1.7 mM sodium L-glutamine [Lonza]).

### SARS-CoV-2–specific T cell response.

To analyze the specific T cell response to SARS-CoV-2, 1.5 × 10^6^ PBMCs were in vitro stimulated for 6 hours at 37°C in R-10 medium with overlapping peptides of protein S (PepMix SARS-CoV-2; Spike Glycoprotein, from JPT Peptide Technologies). In total, 1.5 × 10^6^ PBMCs incubated with the proportional amount of DMSO were included in each experiment as a negative control. The stimulation was performed in the presence of 10 μg/mL of brefeldin A (Sigma-Aldrich) and 0.7 μg/mL of monensin (Golgi Stop, BD Biosciences) protein transport inhibitors, anti–CD107a-BV650 (clone H4A3; BD Biosciences) monoclonal antibody, and purified CD28 (clone CD28.2) and CD49d (clone 9F10) (both from BD Biosciences), as previously described (67, 68). Intracellular cytokines and cytotoxicity markers were analyzed by multiparametric flow cytometry. Specific T cell response was defined as the frequency of cells expressing intracellular cytokines and/or cytotoxicity markers after the stimulation with S peptides minus the levels of this response in the unstimulated condition (background subtraction).

### Monocyte stimulation.

In total, 1 × 10^6^ PBMCs were in vitro stimulated in a 48-well plate for 5 hours at 37°C with 0.5 μL/mL of LPS (Invivogen) in R-10 medium, including 1 × 10^6^ PBMCs without any stimulation as a negative control. A total of 0.7 μg/mL of monensin (Golgi Stop, BD Biosciences) was added to all experimental conditions. Intracellular cytokines were analyzed by flow cytometry.

### mDC stimulation.

PBMCs (0.5 × 10^6^) were in vitro stimulated in a 24-well plate for 24 hours at 37°C with 2 μL/mL of Poly(I:C) (InvivoGen) in R-10 medium. PBMCs (0.5 × 10^6^)incubated without stimulus were included as a negative control. Surface expression of activation markers were analyzed by flow cytometry.

### pDC stimulation culture and quantification of IFN-α production.

Thawed PBMCs (0.5 × 10^6^)were incubated at 37°C and 5% CO_2_ during 18 hours in R-10 medium with or without 1 μM of the TLR9 agonist CpG-A (InvivoGen). After incubation, cells were pelleted, and the supernatants were conserved at –80°C for the subsequent quantification of IFN-α production by ELISA according manufacturer’s instructions (PBL Interferon Source).

### Multiparametric flow cytometry.

In general, for ex vivo phenotyping and functional assays, PBMCs were washed (652*g* for 5 minutes at RT) with phosphate-buffered saline (PBS; Thermo Fisher Scientific). PBMCs were then incubated 35 minutes at RT with a viability marker (LIVE/DEAD Fixable Aqua or Violet Dead Cell Stain; Invitrogen) and all the extracellular antibodies, as mentioned below. PBMCs were washed and fixed and permeabilized with BD Cytofix/CytoPerm (BD Biosciences) at 4°C for 20 minutes or Fixation/Permeabilization Buffer Set (eBioscience) at 4°C for 45 minutes, following the manufacturer’s protocol. Then, cells were stained at 4°C for 30 minutes with intracellular antibodies (as shown below) and washed. Finally, cells were fixed for 20 minutes at 4°C with 4 % paraformaldehyde solution (PFA; Sigma-Adrich).

To assay T cell–specific response, PBMCs were extracellularly stained with LIVE/DEAD Fixable Aqua Dead Cell Stain (Invitrogen), anti–DUMP-channel-BV510 (CD14 clone MφP9, CD19 clone SJ25C1, CD56 clone NMCAM16.2), anti–CD8-APC (clone SK-1), anti–CD3-BV711 (clone SP34-2), anti–CD45RA-FITC (clone L48), anti–CD27-APCH7 (clone M-T271) anti–PD-1–BV786 (CD279, clone EH12-1) (all from BD Bioscience), as well as anti–TIGIT-PerCPCy5.5 (clone A15153G) and anti–LAG-3–BV605 (clone 11C3C65) (both from BioLegend). They were permeabilized and fixed with Cytofix/CytoPerm buffer (BD Bioscience). Cells were intracellularly stained with: anti–IL-2–BV421 (clone MQ1-17H12), anti–IFN-γ–PE-Cy7 (clone B27) (BD Bioscience), anti–TNF-α–AF700 (clone Mab11) (all from BD Pharmingen), and anti–PRF-PE (clone B-D48) (BioLegend). For T cell phenotyping, cells were extracellularly stained with LIVE/DEAD Fixable Aqua Dead Cell Stain (Invitrogen), anti–CD8-PerCP-Cy5.5 (clone SK1), anti–CD45RA-PeCy7 (clone L48), anti–CD3-BV711 (clone SP34-2) (BD Bioscience), anti–HLA-DR–BV570 (clone L243), and anti–CD161-BV421 (clone HP-3G10) (all from BioLegend); they were permeabilized and fixed with fixation/permeabilization buffer (eBioscience) and intracellularly stained with anti-Ki67 FITC (clone 11F6) (BioLegend). T cells were gated based on the CD3 and CD8 expression. Each subset (Memory, CM, EM, and TEMRA) was gated based on CD45RA and CD27 expression (Supplemental Figure 2A).

To assay monocytes functionality, PBMCs were extracellularly stained with LIVE/DEAD Fixable Violet Dead Cell Stain Kit (Invitrogen), anti–DUMP-channel-V450 (CD3 clone SP34-2, CD19 clone HIB19, CD20 clone L27, CD56 clone B159), anti–CD14-BV650 (clone M5E2), anti–CD16-PeCF594 (clone 3G8), and anti–HLA-DR–BV570 (clone L243) (all from BioLegend); they were permeabilized and fixed with BD Cytofix/CytoPerm (BD Biosciences) and intracellularly stained with anti–IL-6–Pe (clone MQ2-6A3), anti–IL-1α–FITC (clone AS5), and anti–TNF-α–AF700 (clone MAb11) (all from BD Biosciences). To assay monocytes phenotyping ex vivo, PBMCs were extracellularly stained with LIVE/DEAD Fixable Violet Dead Cell Stain Kit (Invitrogen), anti–DUMP-channel-V450 (CD3 clone SP34-2, CD19 clone HIB19, CD20 clone L27, CD56 clone B159), anti–CD14-BV650 (clone M5E2), anti–CD16-PeCF594 (clone 3G8), anti–TLR4-BV786 (clone TF901), anti–CD142-Pe (clone HTF-1), and anti–CCR5-APC-Cy7 (clone 2D7/CCR5) (all from BD biosciences), as well as anti–HLA-DR-BV570 (clone L243), anti–TLR2-FITC (clone TL2.1), anti–CD40-APC (clone HB14), anti–CX3CR1-PerCPCy5,5 (clone 2A9-1), anti–CCR2-BV605 (clone K036C2), and anti–CD49d-BV711 (clone 9F10) (all from BioLegend) and anti–CD11b-AF700 (clone VIM12) (Thermo Fisher Scientific). Monocytes were gated based on the CD14 and HLA-DR markers, and nonclassical, intermediate, and classical subsets were gated based on CD14 and CD16 expression (Supplemental Figure 5A).

To assay mDC functionality, PBMCs were extracellularly stained with LIVE/DEAD Fixable Aqua Dead Cell Stain (Invitrogen), anti–CD11c-BV650 (clone B-ly6), anti–HLA-DR-BV570 (clone L243), anti–Lin-2-FITC (CD3 clone SK7, CD19 clone SJ25C1, CD20 clone L27, CD14 clone MφP9, and CD56 clone NCAM16.2), and anti–CD16-BV605 (clone 3G8) (all from BD Biosciences), as well as anti–CD1c-APCCy7 (clone L161), anti–CD141-PeCy7 (clone M80), anti–CD86-BV421 (clone 2331 -FUN-1]), and anti–CD40-APC (clone HB14) (all from BioLegend) and anti–CD83-AF700 (clone HB15) (Invitrogen). They were permeabilized and fixed with BD Cytofix/CytoPerm (BD Biosciences) and intracellularly stained with anti–IDO-Pe (clone eyedio) (eBioscience). For ex vivo DC phenotyping, PBMCs were extracellularly stained with LIVE/DEAD Fixable Aqua Dead Cell Stain (Invitrogen), anti–CD11c-BV650 (clone B-ly6), anti–HLA-DR–BV711 (clone G46-6), anti–Lin-2–FITC, anti–CD16-BV605 (clone 3G8), anti–CCR7-BV786 (CD197) (clone 3D12), anti–CD86-BV421 (clone 2331 [FUN-1]), anti–PD-L1–PeCF594 (CD274) (clone MIH1), and anti–integrin-β7–APC (clone FIB504) (all from BD Biosciences), as well as anti–CD4-PerCPCy5,5 (clone OKT4), anti–CD1c-APCCy7 (clone L161), and anti–CD141-PeCy7 (clone M80) (all from BioLegend) and anti–CD123-AF700 (clone 32703) (R&D Systems) antibodies. Cells were permeabilized and fixed with Fixation/Permeabilization buffer (eBioscience) and intracellularly stained with anti–IDO-Pe (clone eyeido) (eBioscience). DCs were identified by the expression of HLA-DR and the lack of expression of Lin-2. pDCs and mDCS were gated based on the CD123 and CD11c expression, respectively. mDCs subsets were gated using according to CD16, CD1c, and CD141 expression (Supplemental Figure 4A).

Multiparametric flow cytometry were performed on an LRS Fortessa flow cytometer using FACS Diva software (BD Biosciences), acquiring 0.5 to 1 × 10^6^ events. Data were analyzed using the FlowJo 10.7.1 software (TreeStar).

### Quantification of anti–RBD IgG and anti–IFN-α IgG levels.

Anti–RBD IgG SARS-CoV-2 levels were measured by recombinant RBD–specific (rRBD-specific) ELISA as previously described (69, 70, 71, 72). Briefly, Nunc Maxisorp flat-bottomed 96-well plates (Thermo Fisher Scientific) were coated with 1 μg/mL of rRBD protein of the S antigen of SARS-CoV-2 (Sino Biological, 40592-V08H) overnight at 4°C. The following day, plates were blocked with 3% milk in PBS containing 0.05% Tween-20 for 120 minutes at RT. Plasma samples were heat inactivated at 56°C for 20 minutes of complement activity. Human plasma samples were diluted at 1:50, 1:100, 1:200, 1:400, or 1:800 in 1% milk containing 0.05% Tween-20 in PBS and incubated for 90 minutes at RT. Plates were washed 4 times with 0.05% PBS–Tween-20. Human serum standard reference material of anti–SARS-CoV-2 immunoglobulin (first WHO International Standard and International Reference Panel for anti–SARS-CoV-2 immunoglobulin from NIBSC, United Kingdom, NIBSC sample: 20/150) was used as standard curve to titer anti–SARS-CoV-2 IgG antibody in plasma samples. Human serum standard was added to the plates and serially diluted (2-fold dilutions) in 1% milk containing 0.05% Tween-20 in PBS. Pooled plasma samples (NIBSC, United Kingdom, NIBSC sample: 20/142) obtained from healthy blood donors before 2019 was used as negative control plasma. Secondary antibodies, streptavidin-horseradish peroxidase–conjugated mouse anti–human IgG (Hybridoma Reagent Laboratory) was used at a 1:5,000 dilution in 1% milk containing 0.05% Tween-20 in PBS. Plates were washed 4 times with 0.05% PBS–Tween-20. The plates were developed using fast o-Phenylenediamine dihydrochloride Peroxidase Substrate (Sigma-Aldrich); the reaction was stopped using 3 M HCl, and the optical density at 490 nm (OD490) was read on a Multiskan GO Microplate Spectrophotometer (Thermo Fisher Scientific) within 2 hours. Anti–SARS-CoV-2 IgG antibody titers for each donor were calculated as binding antibody units (BAU)/mL according to the manufacturers’ information regarding the WHO Standard and was determined based on sigmoidal dose-response nonlinear regression, 4 parametric logistic (4PL), using GraphPad Prism, version 8.0 (GraphPad Software). For the measurement of anti–IFN-α IgGs, another ELISA assay was performed as previously described (73). In this case, plates were coated with 2 μg/mL of IFN-α2a (Miltenyi Biotec), and plasma samples were diluted 1:10. Eight nonvaccinated healthy donors and 3 patients with severe COVID-19 from a previous study of the group (68) were used as negative and positive controls, respectively.

### DNA extraction and TREC measurement by ddPCR.

The extraction of the genomic DNA from frozen PBMCs was performed using a blood DNA minikit (Omega; Bio-Tek). The DNA concentration was determined by Qubit assay according to the manufacturer’s protocol (Thermo Fisher Scientific).

TRECs were quantified from extracted DNA by ddPCR (Bio-Rad) based on a previously modified method (74). In total, 20 μM of primer for sjTREC (DTF7, 5′→3′: AGGCTCTGTCTAGTGTGATAAC; DTR66, 5′→3′: TGACATGGA GGGCTGAAC), 10 μM of probe (PB1, 5′→3′: 6FAM-TGGGAGTTGGGACCGCCAGAGAGG-BHQ1; SD1, 5′→3′: HEX-CACCCCTCTGTTCCCCACA- BHQ1), and ddPCR Supermix for probes no dUTP (Bio-Rad) were used. The reference gene used was RPP30 (2 copies per cell) (forward, 5′→3′: GATTTGGACCTGCGAGCG; reverse, 5′→3′: GCGGCTGTCTCC ACAAGT; Probe, 5′→3′: VIC-CTGACCTGAAGGCTCT-BHQ1). The ddPCR conditions were: 10 minutes at 95°C, 40 cycles of 30 seconds at 94 °C, 1 minute at 59°C, and 10 minutes at 98°C. Bio-Rad QuantaSoft software v.1.7.4 was used for determining the TRECs/1 × 10^6^ cells.

### Statistics.

Nonparametric statistical analyses were performed using Statistical Package for the Social Sciences software (SPSS 25.0), RStudio Version 1.3.959, and GraphPad Prism version 8.0 (GraphPad Software). Polyfunctionality pie charts were constructed using Pestle version 1.6.2 and Spice version 6.0 (75). Median and IQRs were used to describe continuous variables, and percentages were used to describe categorical variables. The ROUT method was utilized to identify and discard outliers. Differences between aged and young groups were analyzed by 2-tailed nonparametric Mann-Whitney *U* test. The nonparametric Wilcoxon test was used to analyze differences between time points. The Spearman test was used to analyze correlations between variables. For multiple comparisons, Friedman test was applied, including Bonferroni correction. Permutation test was used to assess differences between pie charts using Spice software. Hmisc and corrplot packages were used in R by the Spearman method to calculate correlations between pairs of variables and to plot the correlation matrix figures. Lateral intensity bar from red to blue, next to correlation matrixes, represents the ρ coefficient value of the Spearman test. All differences with *P* < 0.05 were considered statistically significant.

### Study approval.

The study was approved by the Ethics Committee of the Virgen del Rocio University Hospital (COVIMARATON; 0896-N-20). Written informed consent was received from all the participants of the study.

## Author contributions

ERM conceived and designed the research. APG, CGC, IRJ, MDMSS, AMRM, MAMS, MREIB, and ERM participated in sample collection and processing. JV, APG, FJO, CGC, and MRJL performed the experiments. JV carried out the data analysis. JV, SB, LFLC, MREIB, and ERM participated in the interpretation/discussion of the results. JV, APG, and ERM wrote the manuscript. ERM and JV coordinated the project. All authors reviewed and discussed the manuscript.

## Supplementary Material

Supplemental data

## Version 1 08/09/2022

In-Press Preview

## Version 2 09/08/2022

Electronic publication
