# Supplementary material for: Immune defects associated with lower SARS-CoV-2 BNT162b2 mRNA vaccine response in aged people
Source: JCI Insight. 2022 Sep 8;7(17):e161045. doi: 10.1172/jci.insight.161045 (PMC9536264; doi:10.1172/jci.insight.161045)
Supplement: Supplemental data [file jciinsight-7-161045-s092.pdf]

Figure S1

A

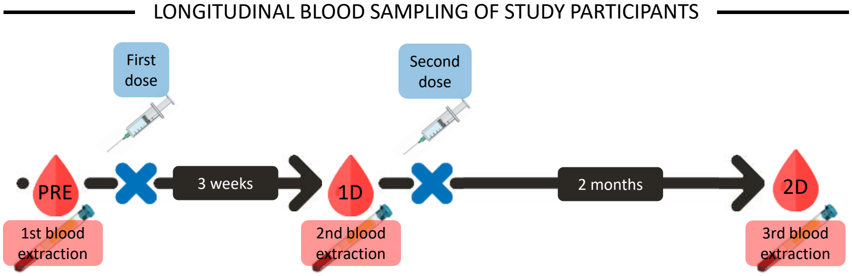

	All participants (n=54)	Elderly participants (n=21)	Young participants (n=33)	p-value
Age (years)	49.5 [28 – 73]	73 [72 – 74]	29 [26 –48.5]	<0.001
Sex (Female sex), n (%)	35 (64.8)	13 (61.9)	22 (66.7)	0.721
Comorbidities, n (%)	26 (48.10)	16 (76.2)	10 (30.30)	0.007
Cardiovascular disease	*10 (18.50)*	*6 (28.6)*	*4 (12.12)*	*0.129*
Cancer	*1 (1.85)*	*1 (4.76)*	*0 (0)*	*0.206*
Thyroid disease	*2 (3.7)*	*1 (4.76)*	*1 (3.03)*	*0.743*
Allergies	*5 (9.25)*	*1 (4.76)*	*4 (12.12)*	*0.363*
Arthritis/Arthrosis	*6 (10)*	*6 (28.6)*	*0 (0)*	*0.001*
Other	*2 (3.7)*	*1 (4.76)*	*1 (3.03)*	*0.743*
None	*26 (48.10)*	*5 (23.8)*	*21 (63.64)*	*0.004*
Unknown	*2 (3.7)*	*0 (0)*	*2 (6.06)*	*0.250*

B

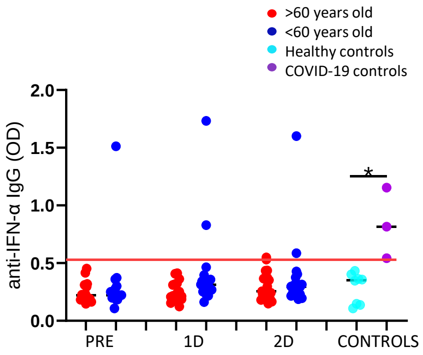

Figure S2

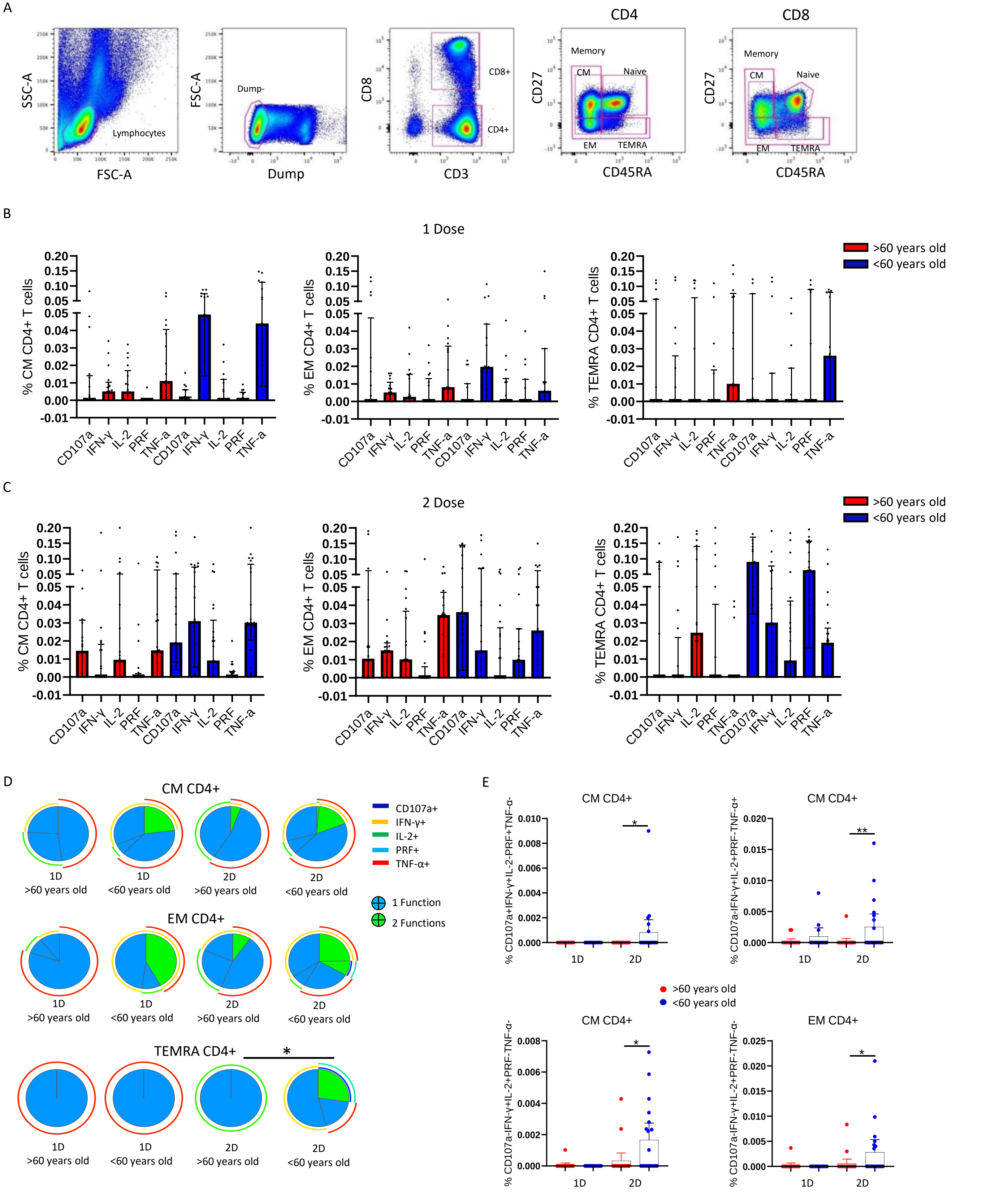
A
CD4
CD8
SSC-A
FSC-A
Lymphocytes
FSC-A
Dump-
Dump
CD8
CD8+
CD4+
CD3
CD27
Memory
CM
Naive
EM
TEMRA
CD45RA
B
1 Dose
>60 years old
<60 years old
% CM CD4+ T cells
% EM CD4+ T cells
% TEMRA CD4+ T cells
CD107a
IFN-γ
IL-2
PRF
TNF-a
C
2 Dose
D
CM CD4+
EM CD4+
TEMRA CD4+
1D
2D
>60 years old
<60 years old
CD107a+
IFN-γ+
IL-2+
PRF+
TNF-α+
1 Function
2 Functions
E
% CD107a+IFN-γ+IL-2-PRF+TNF-α-
% CD107a-IFN-γ+IL-2+PRF-TNF-α+
% CD107a-IFN-γ+IL-2+PRF-TNF-α-
EM CD4+

Figure S3

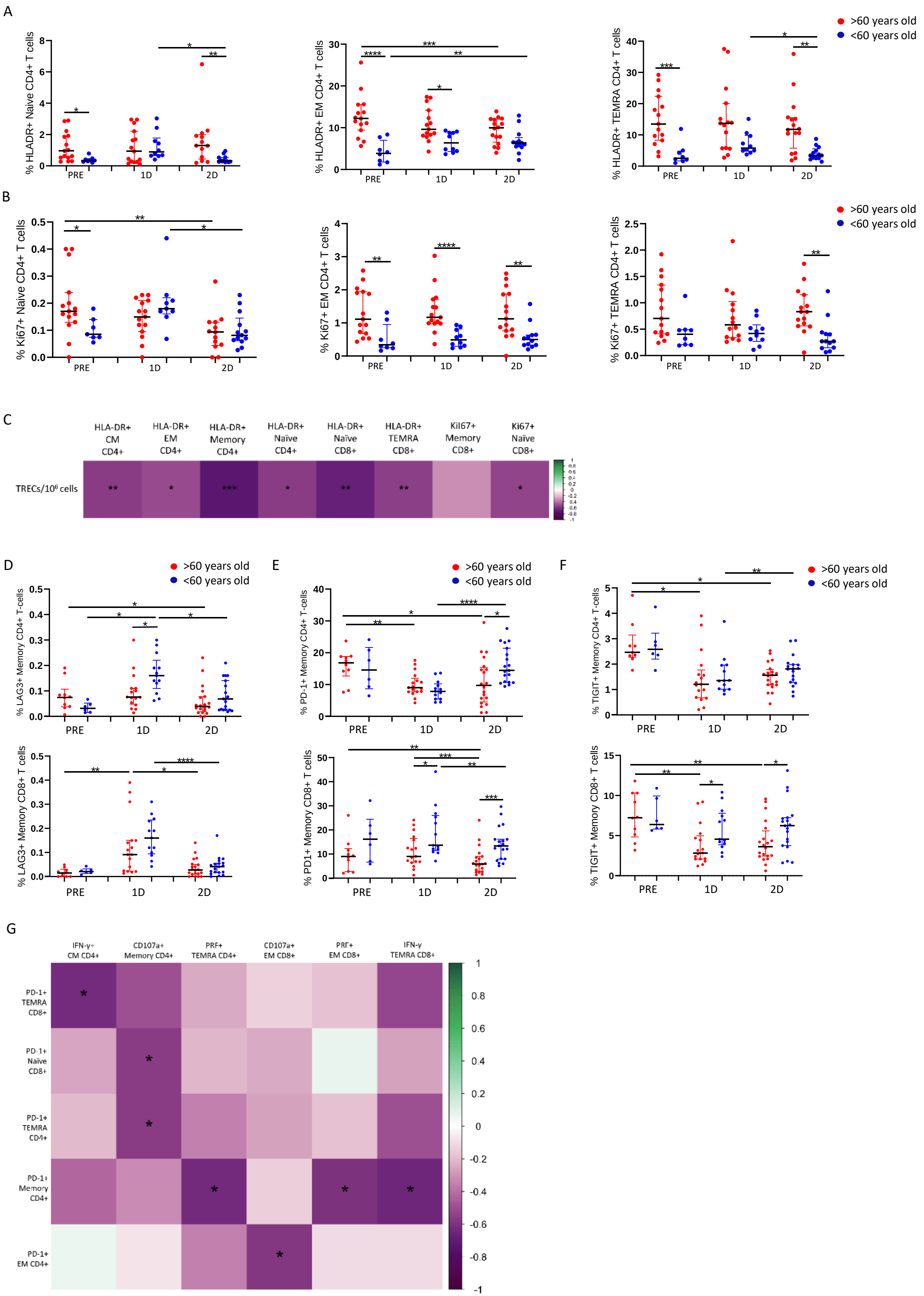
A
% HLADR+ Naive CD4+ T cells
% HLADR+ EM CD4+ T cells
% HLADR+ TEMRA CD4+ T cells
>60 years old
<60 years old
PRE
1D
2D
B
% Ki67+ Naive CD4+ T cells
% Ki67+ EM CD4+ T cells
% Ki67+ TEMRA CD4+ T cells
C
HLA-DR+ CM CD4+
HLA-DR+ EM CD4+
HLA-DR+ Memory CD4+
HLA-DR+ Naive CD4+
HLA-DR+ Naive CD8+
HLA-DR+ TEMRA CD8+
Ki67+ Memory CD8+
Ki67+ Naive CD8+
TRECs/10^6 cells
D
% LAG3+ Memory CD4+ T-cells
% LAG3+ Memory CD8+ T cells
E
% PD-1+ Memory CD4+ T-cells
% PD1+ Memory CD8+ T cells
F
% TIGIT+ Memory CD4+ T-cells
% TIGIT+ Memory CD8+ T cells
G
IFN-γ+ CM CD4+
CD107a+ Memory CD4+
PRF+ TEMRA CD4+
CD107a+ EM CD8+
PRF+ EM CD8+
IFN-γ TEMRA CD8+
PD-1+ TEMRA CD8+
PD-1+ Naive CD8+
PD-1+ TEMRA CD4+
PD-1+ Memory CD4+
PD-1+ EM CD4+

Figure S4

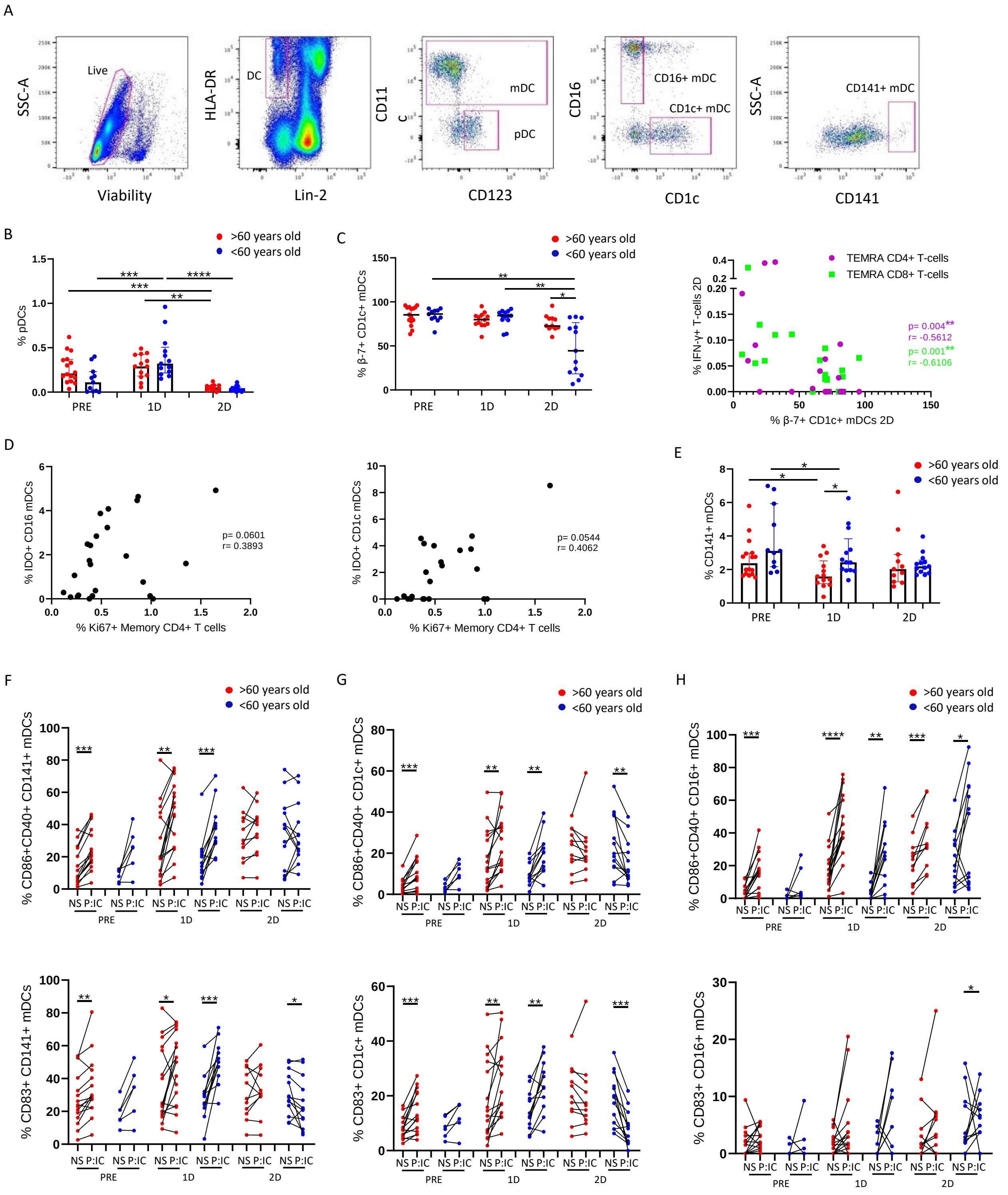
A
SSC-A
Viability
Live
HLA-DR
Lin-2
DC
CD11c
CD123
mDC
pDC
CD16
CD1c
CD16+ mDC
CD1c+ mDC
SSC-A
CD141
CD141+ mDC
B
>60 years old
<60 years old
% pDCs
PRE
1D
2D
C
% β-7+ CD1c+ mDCs
TEMRA CD4+ T-cells
TEMRA CD8+ T-cells
% IFN-γ+ T-cells 2D
% β-7+ CD1c+ mDCs 2D
p= 0.004**
r= -0.5612
p= 0.001**
r= -0.6106
D
% IDO+ CD16 mDCs
% Ki67+ Memory CD4+ T cells
p= 0.0601
r= 0.3893
% IDO+ CD1c mDCs
p= 0.0544
r= 0.4062
E
% CD141+ mDCs
F
% CD86+CD40+ CD141+ mDCs
% CD83+ CD141+ mDCs
NS P:IC
G
% CD86+CD40+ CD1c+ mDCs
% CD83+ CD1c+ mDCs
H
% CD86+CD40+ CD16+ mDCs
% CD83+ CD16+ mDCs

Figure S5

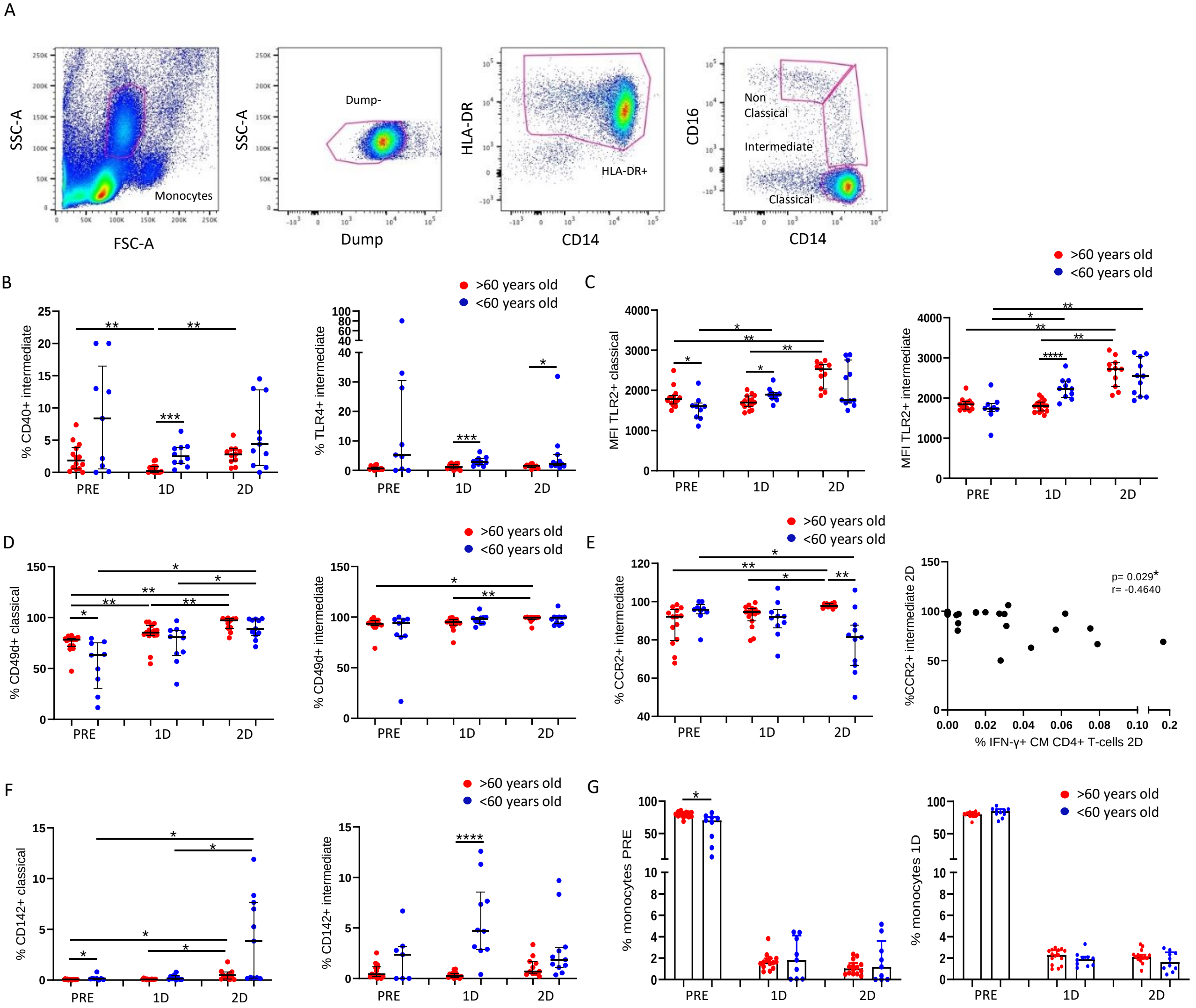
A
SSC-A
FSC-A
Monocytes
Dump-
Dump
HLA-DR
CD14
HLA-DR+
CD16
Non Classical
Intermediate
Classical
B
% CD40+ intermediate
% TLR4+ intermediate
>60 years old
<60 years old
PRE
1D
2D
C
MFI TLR2+ classical
MFI TLR2+ intermediate
D
% CD49d+ classical
% CD49d+ intermediate
E
% CCR2+ intermediate
%CCR2+ intermediate 2D
p= 0.029*
r= -0.4640
% IFN-γ+ CM CD4+ T-cells 2D
F
% CD142+ classical
% CD142+ intermediate
G
% monocytes PRE
% monocytes 1D

Figure S1. Sampling and clinical data of study participants and anti-IFN-α IgG plasma levels

(A) Aged and young donors were vaccinated with two doses of BNT162b2 mRNA vaccine, receiving the second dose three weeks after the first one (top). For this study, peripheral blood samples were extracted from all participants just before SARS-CoV-2 vaccination (PRE), three weeks after the first dose and just before the second one (1D), and two months after the second dose (2D) (top). The table describes the age, sex and comorbidities of studied donors. Variables are expressed as number (n) and percentages (%), and continuous variables are expressed as median with interquartile ranges [IQR]. **(B)** Dot plots showing the optical density (OD) representing anti-IFN-α IgG levels in plasma from >60 years old (red) and <60 years old (blue) participants before SARS-CoV-2 vaccination (PRE), three weeks after the first dose (1D) and two months after the second dose (2D) of vaccination. Eight non-vaccinated healthy donors and three severe COVID-19 patients were used as negative and positive controls, respectively. Mann-Whitney U and Wilcoxon tests were used (n= 54).

Figure S2. SARS-CoV-2 S-specific T cell response in aged and young people

(A) Gating strategy of T cells is shown. Lymphocytes were firstly identified according to their size and complexity (FSC-A and SSC-A) and cells negative for dump channel (viability, CD14, CD19, and CD56) were gated. Then, CD8+ (CD3+ CD8+) and CD4 (CD3+ CD8-) T cells were selected and T cell subsets were identified based on the expression of CD45RA and CD27: naïve (CD45RA+ CD27-) central memory (CM) (CD45RA- CD27+), effector memory (EM) (CD45RA- CD27-) and terminal differentiated effector memory (TEMRA) (CD45RA+ CD27+) cells. Total memory cells (Memory) correspond to the sum of CM, EM and TEMRA T cells. **(B and C)** Bar graphs showing the percentages of CM, EM and TEMRA CD4+ T cells expressing CD107a, IFN-γ, IL-2, PRF and TNF-α after S-specific SARS-CoV-2 stimulation, comparing >60 years old (red) and <60 years old (blue) subjects three weeks after the first dose (B) and two months after the second dose (C) of SARS-CoV-2 vaccine. **(D)** Pie charts representing SARS-CoV-2 S-specific EM, CM and TEMRA CD4+ T cell polyfunctionality. Each sector represents the proportion of S-specific CD4+ T cells producing two (green) or one (blue) functions. Arcs represents the type of function

(CD107a, IFN-γ, IL-2, PRF and TNF-α) expressed in each sector. **(E)** Bar graphs showing the percentage of EM and CM CD4+ T cells expressing different combinations of studied functions (CD107a, IFN-γ, IL-2, PRF and TNF-α) comparing >60 years old (red) and <60 years old (blue) subjects after the first (1D) and the second (2D) dose. Mann-Whitney U, Wilcoxon and Permutation tests were used (n= 41).

Figure S3. T cell homeostasis parameters and its association with SARS-CoV-2 specific T cell response in aged and young people

(A and B) Bar graphs representing the percentage of naïve (left), EM (middle) and TEMRA (right) CD4+ T cells expressing HLA-DR (A) and Ki67 (B) in >60 years old (red) and <60 years old (blue)participants before SARS-CoV-2 vaccination (PRE), three weeks after the first dose (1D) and two months after the second dose (2D) of vaccination. **(C)** Correlation matrix representing associations of TRECs/10^6 cells with the percentages of HLA-DR+ and Ki67+ T cells prior vaccination. **(D-G)** Dot plots representing the percentage of memory CD4+ and CD8+ T cells expressing the immune check points LAG-3 (D), PD-1 (E) and TIGIT (F) in >60 years old (red) and <60 years old (blue)participants at the three follow up time points. **(G)** Correlation matrix representing associations of the percentage of PD-1+ T cells with SARS-CoV-2 S-specific CD4+ and CD8+ T cells expressing IFN-γ and cytotoxicity markers. Mann-Whitney U, Wilcoxon and Spearman tests were used (n= 32).

Figure S4. Dendritic cells phenotype and function before and after SARS-CoV-2 vaccination in aged and young people

(A) Gating strategy of DCs. First live cells were selected and DCs were identified by gating HLA-DR+ and Lineage-2- cells. Then mDCs (CD11c+) and pDCs (CD123+) were selected and mDC subsets were identified according to the surface expression of CD1c, CD16 and

CD141. **(B)** Bar graphs representing the percentages of pDCs in >60 years old (red) and <60 years old (blue)participants before SARS-CoV-2 vaccination (PRE), three weeks after the first dose (1D) and two months after the second dose (2D) of vaccination. **(C)** Dot plots showing the percentage of integrin-β7+ CD1c+ mDCs in old and young participants at the three time points (left) and correlation plot between the percentage of integrin-β7+ CD1c+ mDCs and the percentage of S-specific IFN-γ+ TEMRA CD4+ and CD8+ T cells two months after the second dose (right). **(D)** Correlations between the percentages of IDO+ CD16+ and CD1c+ mDCs with Ki67+ Memory CD4+ T cells in all participants prior vaccination. **(E)** Bar graphs representing the percentages of CD141+ mDCs in >60 years old (red) and <60 years old (blue)participants at the three time points. **(F-H)** Before and after graphs showing the percentages of CD83+ and CD86+CD40+ within CD141+ (F), CD1c+ (G) and CD16+ (H) mDCs without stimulation (NS) or after TLR-3 stimulation with Poly I:C (P:IC) in >60 years old (red) and <60 years old (blue)participants at the three time points. Mann-Whitney U, Wilcoxon and Spearman tests were used (n= 32).

Figure S5. Monocyte phenotype before and after SARS-CoV-2 vaccination in aged and young people

(A) Gating strategy of monocytes. Monocytes were firstly identified according to their size and complexity (FSC-A and SSC-A) and cells negative for dump channel (viability, CD3, CD19, CD20 and CD56) were gated. Then, HLA-DR+ cells were selected and monocyte subsets were identified according to the expression of CD14 and CD16: classical (CD14++ CD16-), intermediate (CD14++ CD16+) and non-classical (CD14+ CD16+). **(B-F)** Dot plots showing the percentages of CD40+ (B, left) and TLR-4+ (B, right) (B), the median fluorescence intensity of TLR-2 (C) and the percentages of CD49d+ (D), CCR2+ (E) and CD142+ (F) monocytes in >60 years old (red) and <60 years old (blue)participants before SARS-CoV-2 vaccination (PRE), three weeks after the first dose (1D) and two months after the second dose (2D) of vaccination. Correlation of the percentage of CCR2+ monocytes with the percentage of IFN-γ+ CM CD4+ T cells after the second dose (E, right). **(G)** Bar graphs representing the percentage of classical, intermediate and non-classical monocytes in >60 years old (red) and <60 years old

(blue)participants before vaccination (left) and after the second dose (right). Mann-Whitney U, Wilcoxon and Spearman tests were used (n= 32).
